# From Water into Sediment—Tracing Freshwater *Cyanobacteria* via DNA Analyses

**DOI:** 10.3390/microorganisms9081778

**Published:** 2021-08-21

**Authors:** Ebuka Canisius Nwosu, Patricia Roeser, Sizhong Yang, Lars Ganzert, Olaf Dellwig, Sylvia Pinkerneil, Achim Brauer, Elke Dittmann, Dirk Wagner, Susanne Liebner

**Affiliations:** 1 GFZ German Research Centre for Geosciences, Section Geomicrobiology, 14473 Potsdam, Germany; sizhong.yang@gfz-potsdam.de (S.Y.); lars.ganzert@uit.no (L.G.); dirkwagner@gfz-potsdam.de (D.W.); sliebner@gfz-potsdam.de (S.L.); 2 Leibniz Institute for Baltic Sea Research Warnemünde, Marine Geology, 18119 Rostock, Germany; patricia.roeser@io-warnemuende.de (P.R.); olaf.dellwig@io-warnemuende.de (O.D.); 3 GFZ German Research Centre for Geosciences, Section Climate Dynamics and Landscape Evolution, 14473 Potsdam, Germany; sytopi@gfz-potsdam.de (S.P.); brau@gfz-potsdam.de (A.B.); 4 Institute of Geosciences, University of Potsdam, 14476 Potsdam, Germany; 5 Institute of Biochemistry and Biology, University of Potsdam, 14476 Potsdam, Germany; editt@uni-potsdam.de

**Keywords:** *Aphanizomenon*, *Planktothrix*, *Snowella*, *cyanobacteria* sedimentation, lake monitoring, sedimentary ancient DNA, sediment traps, environmental reconstruction

## Abstract

Sedimentary ancient DNA-based studies have been used to probe centuries of climate and environmental changes and how they affected cyanobacterial assemblages in temperate lakes. Due to *cyanobacteria* containing potential bloom-forming and toxin-producing taxa, their approximate reconstruction from sediments is crucial, especially in lakes lacking long-term monitoring data. To extend the resolution of sediment record interpretation, we used high-throughput sequencing, amplicon sequence variant (ASV) analysis, and quantitative PCR to compare pelagic cyanobacterial composition to that in sediment traps (collected monthly) and surface sediments in Lake Tiefer See. Cyanobacterial composition, species richness, and evenness was not significantly different among the pelagic depths, sediment traps and surface sediments (*p* > 0.05), indicating that the *cyanobacteria* in the sediments reflected the cyanobacterial assemblage in the water column. However, total cyanobacterial abundances (qPCR) decreased from the metalimnion down the water column. The aggregate-forming (*Aphanizomenon*) and colony-forming taxa (*Snowella*) showed pronounced sedimentation. In contrast, *Planktothrix* was only very poorly represented in sediment traps (meta- and hypolimnion) and surface sediments, despite its highest relative abundance at the thermocline (10 m water depth) during periods of lake stratification (May–October). We conclude that this skewed representation in taxonomic abundances reflects taphonomic processes, which should be considered in future DNA-based paleolimnological investigations.

## 1. Introduction

Anthropogenic environmental changes continue to affect the community dynamics of the ancient and widespread bacterial phylum *cyanobacteria* [1,2]. In some temperate oligo-mesotrophic lakes, warming and prolonged stratification have led to the dominance of toxin-producing species belonging to *Planktothrix*, *Dolichospermum*, and *Microcystis*, despite a reduction in nutrient inputs [3,4]. Due to the ecological impact of *cyanobacteria* in aquatic ecosystems, such as toxic bloom formation [5], their reconstruction from aquatic sediments serving as environmental archives could provide vital information regarding their history and earlier conditions in a water body [6].

Early paleolimnology studies reconstructed the occurrence of *cyanobacteria* in freshwater sediments by targeting their remains, e.g., the akinetes of *Aphanizomenon* and *Anabaena* [7,8] or pigments [9,10,11]. Findings from these earlier studies established our understanding of the long-term survival of *cyanobacteria* remains in sediments and how they could be used to track pelagic dominance of *cyanobacteria* in lakes [9,12], thus establishing a basis upon which molecular-based paleolimnology studies began [13]. 

Combining (paleo)limnology with DNA-based tools has gained popularity in recent times because of the high taxonomic resolution they provide [14]. For example, targeting the 16S rRNA gene revealed a novel pico-cyanobacteria clade in the sediments of a glacial lake [15], and resulted in the identification of higher diatom species diversity in a tropical lake [16]. Targeting of methane-oxidizing genes in bacteria revealed possible methanotrophy in lake sediments [17]. As DNA-based techniques available for paleolimnology studies continue to advance, the amount of new information on ancient organisms has increased [14,18], as has the demand for paleolimnological studies on past abiotic–biotic relationships [19]. 

With the increase in sedimentary ancient DNA (sedaDNA) studies in the last decade, there is a need to verify the congruence between sediment-archived DNA and the pelagic communities that acted as seeds, as well as to assess potential taphonomic processes, i.e., alteration or deterioration of DNA during transport and at the sediment–water interface under prevailing environmental conditions [19,20,21]. There is a need to verify congruence, in particular, because taphonomic processes could lead to the underrepresentation of certain groups in sedimentary records [22,23,24]. A common approach used to substantiate sedaDNA information has focused on comparing molecular data obtained from the water column and short sediment cores (up to 30 cm) for planktonic protists [20]. Recently, Gauthier et al. used DNA-based methods on sediment trap and water samples to trace eukaryotic communities that deposit in sediment traps from the water column [25]. For cyanobacteria, consistency between historical microscopic data and sedimentary DNA have been shown [26].

To date, our understanding of how representative sediment-deposited *cyanobacteria* are for those in the water column is, however, still restricted to a few studies. Among these, lakes whose sediments serve as highly resolved paleo-archives are not well represented. Therefore, this study investigated the congruity between pelagic and sediment-deposited *cyanobacteria* communities in Lake Tiefer See, a well-studied climate archive and monitoring site located in northeastern Germany. Specifically, we tracked changes in cyanobacterial sedimentation by generating monthly DNA-based high-throughput sequence and abundance data from the water column (covering the epi-, meta-, and hypolimnion), sediment traps (meta- and hypolimnion), and surface sediments (0–2 cm). We used a highly resolved dataset to assess the suitability of sediment-deposited cyanobacterial assemblages as proxies for lake internal physicochemical parameters by correlating *cyanobacteria* dynamics with daily to monthly resolved water column temperature, pH, dissolved oxygen (DO), turbidity, nitrates, and total dissolved phosphorus (TDP).

## 2. Materials and Methods

### 2.1. Study Site

**Figure 1:**
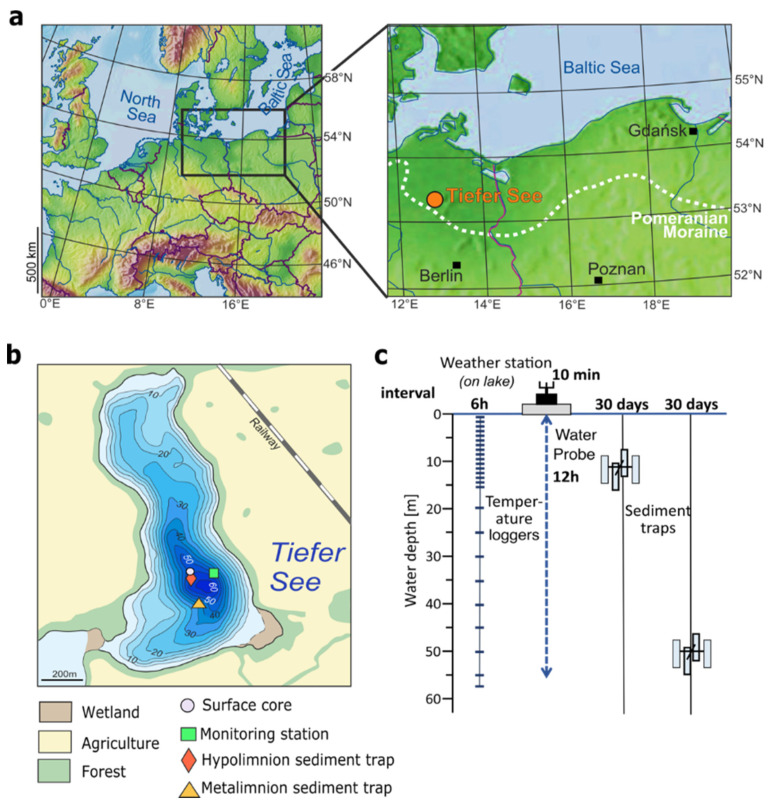
Location of the study site and information on the monitoring setup: (**a**) The location of Lake Tiefer See near Klocksin (TSK, northeastern Germany); (**b**) A bathymetric map of Lake Tiefer See showing the sites of climate monitoring stations, water sampling, sediment traps, and surface core extraction, and (**c**) A scheme of lake monitoring setup showing depths of water column, temperature loggers, and depths at which the sediment traps are anchored.

The hard-water oligo-mesotrophic Lake Tiefer See (near Klocksin; 53°35.5′ N, 12°31.8′ E; 62 m a.s.l.) is part of the southern Baltic Lake District and was formed during the final stage of the last glacial period (~13,000 years ago; Figure 1a,b; Supplementary Information). Lake Tiefer See is part of a subglacial gully system in a morainic terrain located in Nature Park ‘Nossentiner/Schwinzer Heide’. Lake Tiefer See has a surface area of approximately 0.75 km^2^, a catchment area of approximately 5.5 km^2^ dominated by glacial till and a maximum depth of 62 m with no major inflows or outflow. Lake Tiefer See has been the focus of an extensive and high-resolution climate monitoring program since the beginning of the last decade [27]. The lake is well-preserved and seasonally laminated sediments have aided climate reconstructions spanning the last 6000 years [28].

### 2.2. Collection of Water, Sediment Trap Material, and Sediment Cores 

Water samples were collected monthly between January 30 and November 28 in 2019 at the floating weather monitoring station, located at the maximum depth of the lake (62 m; Figure 1b). The sampled water depths were 1, 3, 5, 7, 10, 15, 20, 40, 45, and 50 m. A sample volume of 250 mL of lake water was collected for the upper 1, 3, and 5 m water depths and pooled (750 mL), representing the epilimnion. Likewise, 375 mL volumes were collected for the lower 45 and 50 m water depths and pooled (750 mL), representing the bottom waters. For each of the other 7, 10, 15, 20, and 40 m water depths, 750 mL water samples were collected. Samples were collected in sterile glass bottles (Schott Duran^®^, Mitterteich, Germany), filtered within 24 h after fieldwork using 0.2 µm cellulose filters (Sartorius AG, Göttingen, Germany). Filters were stored at −20 °C until nucleic acid extraction. 

Suspended particulate matter was collected using four-cylinder traps (KC Denmark A/S, total active area 0.0163 m^2^ for the four cylinders) at two depths in the water column (Figure 1c). One trap was anchored in the metalimnion (12 m water depth), and the second trap was secured in the hypolimnion (55 m water depth). For both depths, the traps were emptied monthly between April and November 2019, i.e., the annual interval of increased lake productivity. The trapped material was transferred into 2 L plastic bottles, which were allowed to settle overnight at 4 °C. About 1200 mL of water was discarded from the bottles, and the remaining suspension was transferred into sterile 50 mL Falcon tubes (Fischer Scientific GmbH, Schwerte, Germany). Tubes containing suspended particulate matter were then centrifuged at 4000× *g* for 5 min. Subsequently, the supernatant was discarded, and the pellet was stored in 50 mL Falcon tubes at −20 °C until nucleic acid extraction. 

A surface sediment core (TSK19-SC6; 115 cm length) was collected on 29 August 2019, from the point of maximum water depth (62 m) using a 90 mm UWITEC piston corer. The temperature of the upright positioned core was maintained at 4 °C during transportation and short-term storage at the facilities of the GFZ Potsdam. To preserve the uppermost varves, the core was allowed to dry in a vertical position at 4 °C for approximately 2 weeks before longitudinal splitting. After splitting the core, one half was lithologically described. A triplicate 0–2 cm subsample of the other core half was stored in sterile 15 mL Falcon tubes at −20 °C until nucleic acid extraction. All core handling was performed under clean conditions in a room where no molecular biological work had been previously conducted to avoid contamination.

### 2.3. Lake Physicochemical Properties

Water column temperature, pH, DO, turbidity, and chlorophyll-a (Chl*a* which we used as an indicator of phytoplankton biomass production) were measured using a multi-parameter water quality probe (YSI 6600 V2, Yellow Springs, Greene, OH, USA), in 1 m steps and 12 h resolution. Owing to technical problems, in February data were only collected on 2 d (1st and 28th), and in March no data were recorded between the 2nd and 10th. Water temperature was additionally measured using 26 stationary data loggers (HOBO Water Temp Pro v2, Onset USA) in 1 m steps from 0 to 15 m and 5 m steps from 15 to 55 m water depth (Figure 1c). Water samples for nitrate (NO_3_^–^) and total dissolved phosphorus (TDP) analyses were also collected monthly, in parallel with those for molecular analysis. Water samples for total sulfide were collected from the hypolimnion in October and November. Nitrate was measured by suppressed ion chromatography using a SeQuant SAMS anion IC suppressor (EMD Millipore, Billerica, MA, USA), an S5200 sample injector, a 3.0 × 250 mm LCA 14 column, and an S3115 conductivity detector (all Sykam, Fürstenfeldbruck, Germany). The TDP was measured by inductively coupled plasma optical emission spectrometry (ICP-OES; iCAP 7400, Duo, Thermo Fisher Scientific, Berlin, Germany) using external calibration and Sc as the internal standard. 

Sediment fluxes (in g m^−2^ d^−1^) were determined from the weighted and freeze-dried trapped sediment material. Total carbon (TC), total organic carbon (TOC, and total nitrogen (TN) contents were measured continuously at 1 cm increments from bulk samples as described by Dräger et al. [28] using the elemental analyzers NC2500 and EA3000-CHS Eurovectors, respectively. Briefly, TIC and TN were measured from 5 mg of sediment in tin capsules, while TOC was examined by decalcifying 3 mg of sediment in Ag capsules by treating with 3% HCl, 20% HCl, and drying at 75 °C. The TOC and TN values were used to calculate the atomic C:N ratio. Calcium carbonate (CaCO_3_) was estimated after obtaining the total inorganic carbon content (TIC = TC–TOC) and multiplying by a factor of 8.33, which is the percentage of molecular weight in inorganic carbon in the calcium carbonate structure.

### 2.4. Molecular Analyses

All monthly water samples plus two depths of sediment traps in four biological replicates and the surface sediments were processed independently from each other and at different times to avoid cross-contamination. Due to a mixed water column in January and February, water samples for molecular analyses from 1, 3, 5, 7, and 10 m water depths were pooled and reported as the mean depth (5 m). Water depths of 40, 45, and 50 m were equally pooled and reported as the mean depth (45 m). In March, when temperatures began to increase, four depths in the water column were reported as follows: 5 (1, 3, 5, and 7 m pooled), 10, 20, and 45 m (40, 45, and 50 m pooled). Genomic DNA, PCR amplification, library preparation and bioinformatics were carried out as described in Supplementary Information. Briefly, DNA from water samples was extracted from the filters using the DNeasy PowerWater Kit (QIAGEN, Hilden, Germany) following the manufacturer’s specifications. The DNA from the homogenized sediment trap samples and surface sediment samples was extracted from approximately 0.75 g using the DNeasy PowerSoil Kit (QIAGEN, Hilden, Germany). DNA concentrations for both water, sediment trap, and surface sediment samples were measured using a Qubit (2.0) Fluorometer (Invitrogen, Carlsbad, CAL, USA) highly selective for double-stranded DNA following the manufacturer’s instructions (Qubit ds-DNA HS Assays, Invitrogen, Carlsbad, CAL, USA). Total *cyanobacteria* were quantified with a SYBR Green quantitative PCR (qPCR) assay that specifically amplified the cyanobacterial 16S rRNA-ITS (internal transcribed spacer) region. Each water sample was quantified in triplicates and the four biological replicates (i.e., four cylinders) from the monthly meta- and hypolimnion sediment trap samples were each quantified separately in triplicates. The values were calculated following Savichtcheva et al. [29], and expressed as cyanobacterial abundance normalized to extracted DNA (copies ng^−1^ DNA).

The PCR for the Illumina high-throughput sequence libraries was conducted using the cyanobacteria-specific primers CYA359F (5′-CGGACGGGTGAGTAACGCGTG-3′) and CYA784R (5′-ACTACWGGGGTATCTAATCCC-3′) [30] which amplify a >400 nt long fragment of the V3–V4 regions of the 16S rRNA gene. The primers had unique tags (Table S1) that served to differentiate the samples. The PCR products were purified with the Agencourt AMPure XP kit (Beckman Coulter, Brea, CA, USA) and quantified using a Qubit (2.0) fluorometer (Invitrogen). Equimolar concentrations of all samples, including two negative purified PCR controls, were pooled into two multiplex libraries (*n* = 160 samples, including 78 samples and 2 controls per library), which were paired-end sequenced (2 × 300 bp) on an Illumina MiSeq system (Eurofins Scientific; Constance, Germany).

### 2.5. Bioinformatics and Sequence Processing

Sequencing data and metadata are deposited in the European Nucleotide Archive (ENA) under BioProject accession number PRJEB40406 and sample accession numbers ERS5083533—ERS5083564 (trap material and surface sediment samples) and ERS5083566—ERS5083644 (water samples) and processed as described in Figure S1. Briefly, the obtained 14,649,824 sequence reads were quality checked on a raw FASTQ file with FastQC v0.11.8 [31], on a local machine. The reads were then demultiplexed using the make.contigs function in Mothur (v.1.39.5) [32]. Based on the report files, the sequence identifiers were retrieved for those sequences with minimum overlap (length >25), maximum mismatches (<5), and the maximum number of ambiguous bases of zero (which means there was no base marked with N′). The 160 samples resulted in a total of 7,297,946 denoised and error-corrected sequences that DADA2 [33] inferred in 2538 ASVs. We filtered out non-cyanobacteria ASVs, chloroplasts, rare taxa, and negative controls. In total, the filtered dataset comprised 2,031,142 sequence reads in 559 ASVs assigned to photosynthetic *cyanobacteria* and distributed across 64 samples (Table S1). Of the 559 ASVs, 19 were assigned to the order level, 61 to the family level, and 479 to the genus level (86% of all *cyanobacteria* ASVs; 2,025,258 read counts). We presented the distribution of shared and unique ASVs among the sample types in a Venn diagram (Figure S1). 

### 2.6. Data Treatment and Statistics

To compare differences in *cyanobacteria* assemblage from water, sediment traps, and surface sediments, we transformed ASV absolute read counts into relative abundances. To simplify the presentation of the *cyanobacteria* assemblage data, the mean relative abundances for each water (3, 5, 7, 10, 15, 20, and 45 m) and sediment trap sampled (meta- and hypolimnion) depths were merged as shown in bubble plot in Figure 3a. In Figure S3a, we present the highly-resolved *cyanobacteria* composition from all sample matrices. Bubble plots were plotted using the free software tool (http://shiny.raccoome.de/bubblePlot/, accessed on 20 August 2021). To compare the differences in *cyanobacteria* abundance (qPCR) between the sample matrices, the mean copies ng DNA^−1^ for each sampled water depth (3, 5, 7, 10, 15, 20, and 45 m) was calculated. For the sediment traps, the mean of the four biological replicates of each monthly meta- and hypolimnion trap was calculated, followed by calculating the mean of all months (8 months) for the meta- and hypolimnion trap as shown in Figure 3b. In Figure S3b, we present the highly-resolved *cyanobacteria* abundance data from all sample matrices. 

Alpha and beta diversity estimations, ANOVA, and Tukey’s pairwise test for alpha diversity boxplots, correlations of the ASV composition with physicochemical parameters, and similarity percentage (SIMPER) analysis, as well as multivariate permutational analysis of variance (PerMANOVA) were performed using the PAST v4.01 software [34]. Prior to alpha diversity (richness, Shannon diversity and Pielou’s evenness), the ASV read counts were rarefied to account for differences in sequencing depth (2200 read counts per sample) using the “rtk” package in R [35]. Since the biological triplicates of the surface sediment samples are from one core, we pooled them into the mean of one sample before calculating alpha and beta diversity. The water column samples were further grouped into thermal stratification zones, that is, epi-, meta-, and hypolimnion, whereas sediment trap samples were grouped into meta- and hypolimnion, and the surface 0–2 cm of the sediment. A one-way ANOVA was used to test for significant seasonal change in species richness and evenness within each stratification zone followed by a Tukey’s test. *cyanobacteria* absolute read counts were Hellinger-transformed prior to beta diversity analysis and the subsequent PerMANOVA test [36]. Clustering patterns of the *cyanobacteria* community from all sample matrices were assessed using Bray–Curtis dissimilarity in a non-metric multidimensional scaling analysis (NMDS). The PerMANOVA analysis was then used to test for significant community differences within and among the samples. SIMPER analysis, also based on Bray–Curtis dissimilarity, was used to calculate the taxa similarity contributions among the sample types. The environmental data from water and sediment traps were standardized by subtracting the mean and dividing by the standard deviation (z-score) prior to principal component analyses (PCAs) based on the Euclidean distance. Due to the very distinct density and resolution data, PCAs were performed for all physicochemical water parameters and trapped material composition data to assess similarities within the sample types (Figure S2). To control for the effect of confounders in the explanatory variables dataset, collinearity was tested with a variance inflation factor (VIF) using the “vif.cca” function in vegan in R [37]. Explanatory variables were then additively tested until only those with a VIF score <10 remained. The significant subset of the explanatory variables for community composition was determined via forward selection using the function “ordiR2step” function in vegan. A rank-based Spearman correlation coefficient was used to calculate the correlations of cyanobacterial community data from the water column and hypolimnion sediment trap (relative abundances of total and most abundant ASVs from all samples, 16S rRNA-ITS gene abundance, and Shannon diversity indices) to a significant subset of physicochemical parameters (temperature, pH, DO, turbidity, NO_3_^–^, and TDP) with the Bonferroni *p*-value correction. The physicochemical parameters and cyanobacterial community data were the explanatory and response variables, respectively. 

## 3. Results

### 3.1. Physicochemical Properties of Pelagic Water and Trap Material

**Figure 2:**
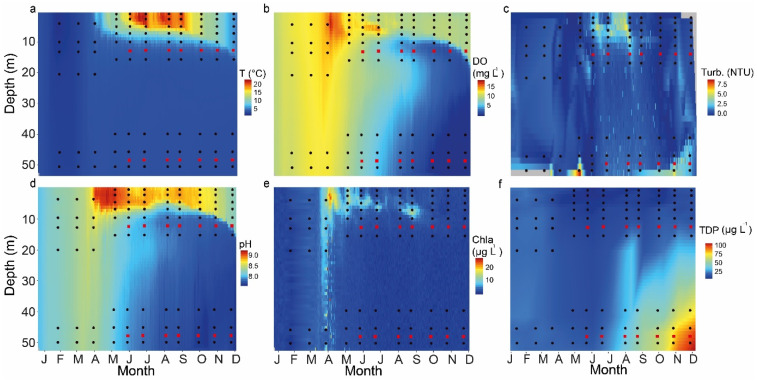
Physicochemical parameters from Lake Tiefer See: Heatmaps of physicochemical parameters for 2019 measured using a multi-parameter water quality probe in the water column of Lake Tiefer See showing (**a**) Temperature (T); (**b**) Dissolved oxygen (DO); (**c**) Turbidity (Turb.); (**d**) pH; (**e**) Chlorophyll-a (Chl*a*), and (**f**) Total dissolved phosphorus (TDP). Data are interpolated for 1 d and 1 m water depth. Black circles and red squares represent the depths and timing of sampling of water and sediment traps, respectively.

Temperature and DO in the water column of Lake Tiefer See showed that thermal stratification began in early April 2019 and ended in late November, with a thermocline between 7 and 13 m water depth (Figure 2a,b). Oxygen depletion in the bottom water began 3 weeks after thermal stratification and the onset of pelagic productivity, as revealed by Chl*a* and DO (Figure 2b,e). The DO reached minimum values (~0.67 mg L^−1^) at 40–50 m water depth between October and December (Figure 2b). In October and November 2019, total sulfide concentrations were found to gradually increase below 53 m water depth, from ~2 µmol L^−1^ to ~45 µmol L^−1^, respectively (Table S4). Lake Tiefer See developed a zone of a metalimnetic oxygen minimum between 10 and 12 m from June to September. Turbidity reached higher values between 5 and 6 NTUs in summer in the upper water column (down to 10 m) because of bio-productivity and calcite formation (Figure 2c). In the hypolimnion, turbidity values reached 6 NTUs, because of resuspension in spring and autumn (April and September). Pelagic nitrate values ranged between 1 and 2 µg L^−1^, except in October and November, when they only reached 0.2 µg L^−1^ (Table S5). The values of TDP (Figure 2f) were generally higher in the hypolimnion, gradually increasing from July through November, reaching up to 67 µg L^−1^ in the bottom waters. In contrast, the TDP values in the epilimnion ranged between 8 and 15 µg L^−1^, except in January and February, when the lake was in an isothermal state and the TDP values were between 20 and 25 µg L^−1^ throughout the mixed water column. The overall pattern of seasonal physicochemical changes and the TDP distribution in Lake Tiefer See throughout the year agrees with the behavior in the previous 5 years [27].

**Figure 3:**
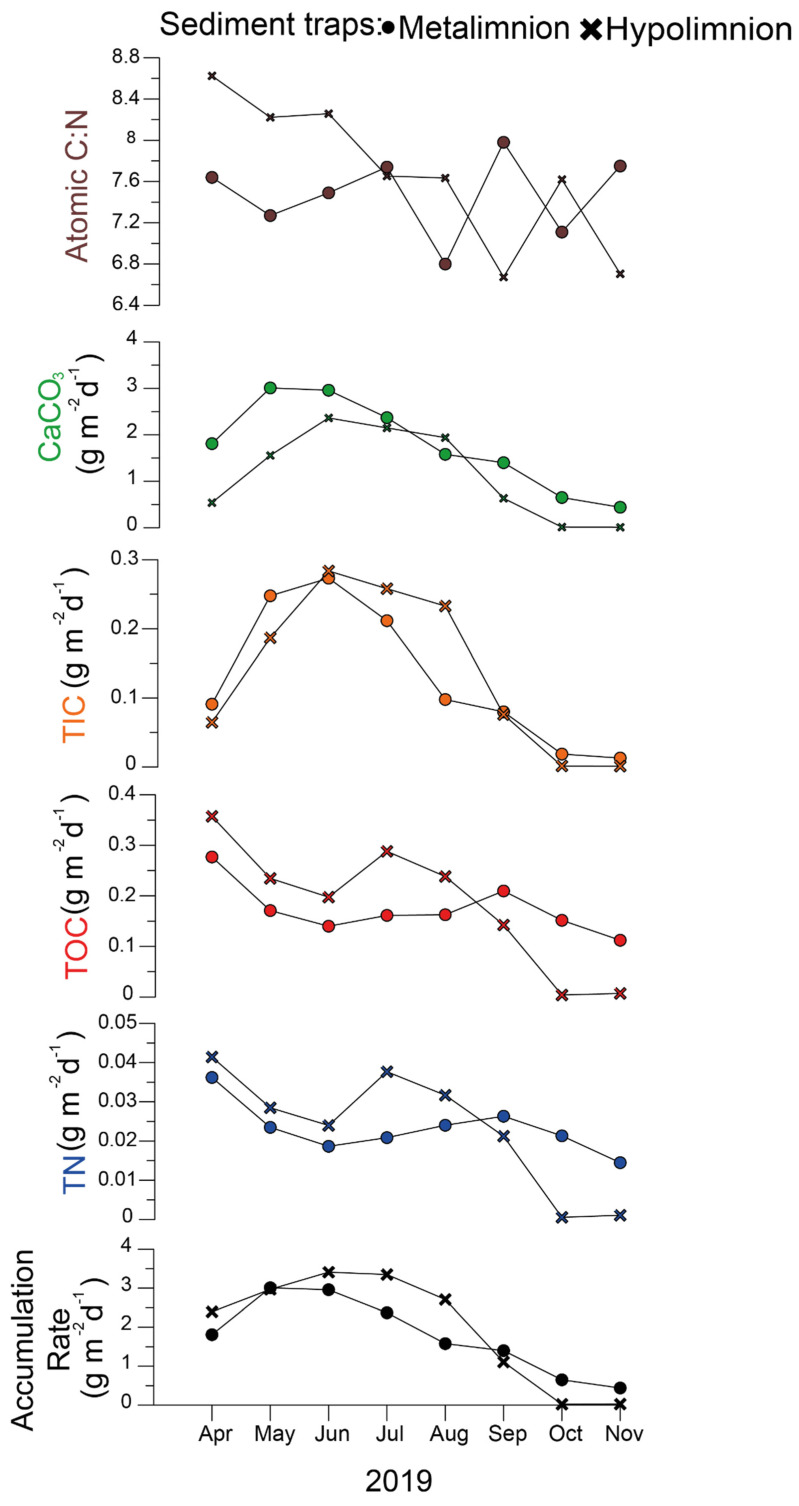
The results of geochemical analysis carried out on sediment trap samples from Lake Tiefer See showing sediment accumulation rate, total nitrogen (TN), total organic carbon (TOC), total inorganic carbon (TIC), calcium carbonate (CaCO3), and atomic TOC and TN ratio (atomic C:N).

During the 1-year cycle, the overall suspended matter accumulation in the metalimnion sediment trap peaked from May to June at 3 g m^−2^ d^−1^. From October to November, particulate deposition at a water depth of 12 m decreased to ~1 g m^−2^ d^−1^ (Figure 3). In the hypolimnion, maximum particulate deposition was reached between June and July, with 4 g m^−2^ d^−1^ (Figure 3). The composition of the trapped material showed an overall contrasting value in the atomic C:N ratio during summer between the meta- (mean 7.5) and hypolimnion (mean 8.4), except in July when the ratio in both meta- and hypolimnion traps was 7.6 (Figure 3). In October and November, TOC, and TN fluxes at the bottom waters (hypolimnion traps) were practically absent, although fluxes of 0.2 g m^−2^ d^−1^ TOC and 0.02 g m^−2^ d^−1^ TN were measured in the metalimnion trap (Figure 3).

Principal component analyses (PCAs) showed that in the water column, the variance was mostly explained by Chl*a*, DO, temperature, and turbidity, with 64% of the variance distributed along the two main principal components, PC1 (44%) and PC2 (20%; Figure S2a). From the trapped sediment composition data, the two main principal components together explained 77% of the total variance, and PC1 accounted for 59% of the variance (Figure S2b). The variables TN, TOC, and sediment deposition rate mainly explained the variance among these samples.

### 3.2. Cyanobacteria Community Structure 

**Figure 4:**
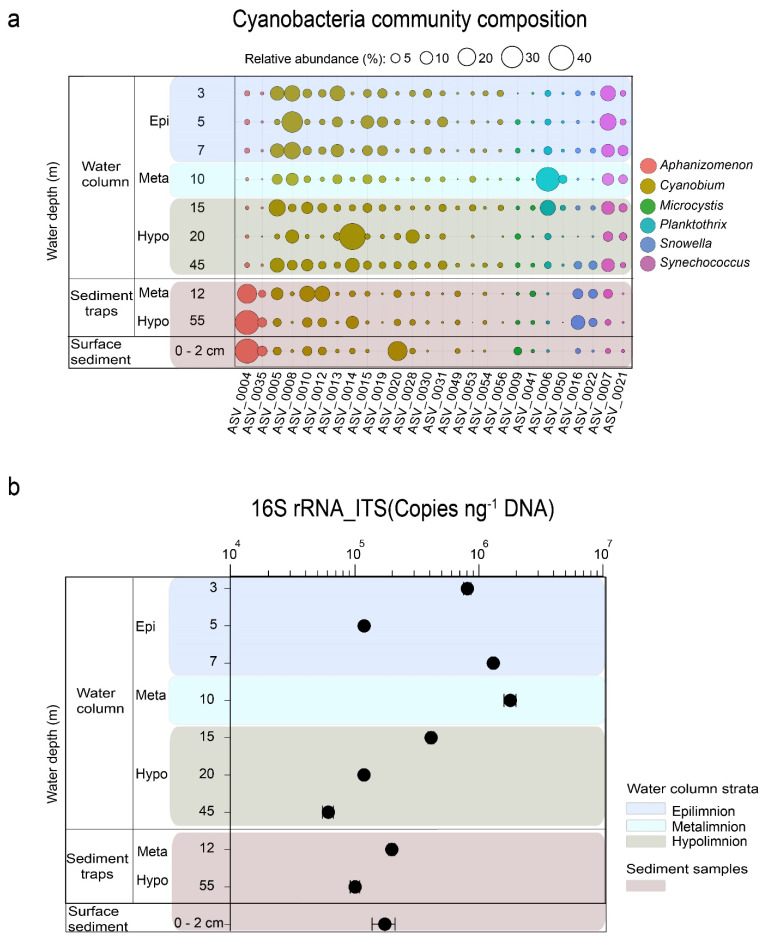
*Cyanobacteria* community composition and abundance in Lake Tiefer See water, trapped material, and sediments: (**a**) Bubble plot showing mean relative abundances for 2019 of the most abundant *cyanobacteria* amplicon sequence variants (>1% reads) in the water column, sediment traps and surface sediments, and (**b**) Mean cyanobacterial abundance for 2019 in the water column, sediment traps, and surface sediments quantified via qPCR with primers that amplified part of the 16S rRNA internal transcriber spacer (ITS) region of *cyanobacteria* (data were normalized to ng DNA extracted). Epi = epilimnion, meta = metalimnion, and hypo = hypolimnion.

Of the 559 total photosynthetic *cyanobacteria* ASVs in this study, only 26 had a relative abundance of ≥1% in the water column, sediment trap material, and surface sediment samples (Figure 4a; Figure S5). They included 16 ASVs assigned to the unicellular pico-*Cyanobium* (order Synechococcales), two ASVs assigned to *Aphanizomenon* (Nostocales), *Planktothrix* (Oscillatoriales), *Snowella*, *Synechococcus* (Synechococcales), and *Microcystis* (Chroococcales). The abundance of the 26 ASVs varied among the sample types. The ASVs 0010 and 0012 assigned to *Cyanobium* and all ASVs assigned to *Aphanizomenon* were more abundant in trapped material (≥10%) than in the corresponding zone in the water column (≤5%). Within this group of ASVs, ASV0004 assigned to *Aphanizomenon* was the most dominant, with ≥30% relative abundance. Conversely, ASVs assigned to *Planktothrix*, ASV0021 assigned to *Synechococcus*, and ASVs 0008, 0013, 0015, 0019, and 0031 assigned to *Cyanobium* were more abundant in the water column (≥10%) but did not commensurate in the sediment traps. In this group of ASVs, ASV0006 assigned to *Planktothrix* was the most prominent, but despite having a maximum abundance (>40%) in the water column in August and October (10 and 15 m), its relative abundance was low (<2%), even in the metalimnion trap at ~12 m water depth (Figure 4a; Figure S3). Additionally, we observed some taxa with comparable abundances across the different water and sediment matrices, including ASVs assigned to *Microcystis*, *Snowella*, ASVs 0028, 0054 (<10%), and ASVs 0005 and 0014, assigned to *Cyanobium* (Figure S3).

#### Cyanobacteria Abundance

In the water column, the highest mean abundance of the cyanobacterial 16S rRNA-ITS gene copy numbers was recorded in the metalimnion (10 m, *n* = 9, 1.8 × 10^6^ copies ng^−1^ DNA; Figure 4b). The mean abundances in the epi- and hypolimnion of the water column were 9.8 × 10^5^ (*n* = 19) and 2.3 × 10^5^ (*n* = 19) copies ng^−1^ DNA, respectively. The mean abundance of the cyanobacterial 16S rRNA-ITS gene copy numbers in the water column was higher (7.9 × 10^5^ copies ng^−1^ DNA) than those of the trapped (1.49 × 10^5^ copies ng^−1^ DNA) and surface (6.94 × 10^5^ copies ng^−1^ DNA) sediments, respectively. The mean abundance of cyanobacterial 16S rRNA-ITS gene copy numbers was 2.2 × 10^5^ and 1.4 × 10^5^ copies ng^−1^ DNA in the meta- and the hypolimnion sediment traps, respectively. 

### 3.3. Alpha and Beta Diversity 

**Figure 5:**
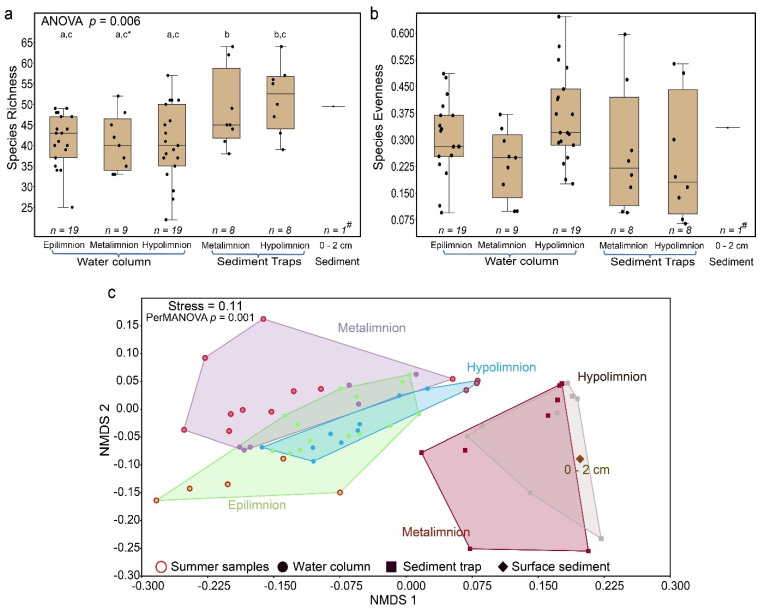
Alpha and beta diversity of *cyanobacteria* communities in Lake Tiefer See: Alpha diversity of *cyanobacteria* community composition in the water column, trap, and surface sediments where (**a**) ANOVA test for equal means on species richness followed by Tukey’s pairwise tests; a = non-significant zones in water column, b = non-significant zones in sediment traps, c = significance in hypolimnion traps, and * = marginal significance. # = one sample representing the mean of biological triplicates; (**b**) ANOVA test for equal means on Pielou’s species evenness among the groups was not significant *p* = 0.07, and (**c**) Visualization of beta diversity via a non-metric multidimensional scaling (NMDS) of *cyanobacteria* community composition in the water column, trap material, and surface sediments based on Bray–Curtis dissimilarity.

Mean *cyanobacteria* species richness was higher in the sediments (trapped and topmost *n* = 17, mean = 50) than in the water column (*n* = 47, mean = 41; Figure 5a). A one-way analysis of variance (ANOVA) test revealed significant variation in *cyanobacteria* species richness between the water (epi-, meta-, and hypolimnion) and sediment traps (meta- and hypolimnion; *p* = 0.006; Figure 5a, Table S6). A subsequent Tukey’s pairwise test showed that richness did not significantly differ between the meta- and hypolimnion traps, as well as among the distinct lake strata in the water column (*p* > 0.05). The difference in richness between the hypolimnion sediment trap and metalimnion of the water column was marginal (*p* = 0.04). The *cyanobacteria* subpopulations among the sample types did not significantly vary in evenness (*p* > 0.05; Figure 5b). 

Bray–Curtis dissimilarity in non-metric multidimensional scaling (NMDS) was used to visualize the beta diversity patterns among the sample types (Figure 5c). The NMDS revealed cyanobacterial community clusters of trap material and surface sediments that were distinct from those of the water column. Within the water column, the overlapping water samples likely resulted from homogenous water when the water column was mixed (Figure 5c). The solitary samples separated from the overlap represent the summer months where a stronger difference in community and environmental parameters occurred because of thermal stratification. A one-way permutational analysis of variance (PerMANOVA) test revealed that there was no significant difference between the meta- and hypolimnion subpopulations of the sediment traps (*p* = 1). The epi- and hypolimnion subpopulations of the water column also did not differ from each other (*p* = 0.08). Similarly, the relative abundance of the cyanobacterial subpopulation from the surface sediment (0–2 cm) was not different from those of the water column (*p* > 0.7) and from those of the sediment traps (*p* = 1; Table 1). A similarity percentage analysis (SIMPER; Table S3) on the ASVs >1% revealed that ASV0004 assigned to *Aphanizomenon* was the strongest (9.34%) contributor to community similarity across pelagic, trapped, and surface sediments.

### 3.4. Correlation of Biotic with Physicochemical Properties

**Figure 6:**
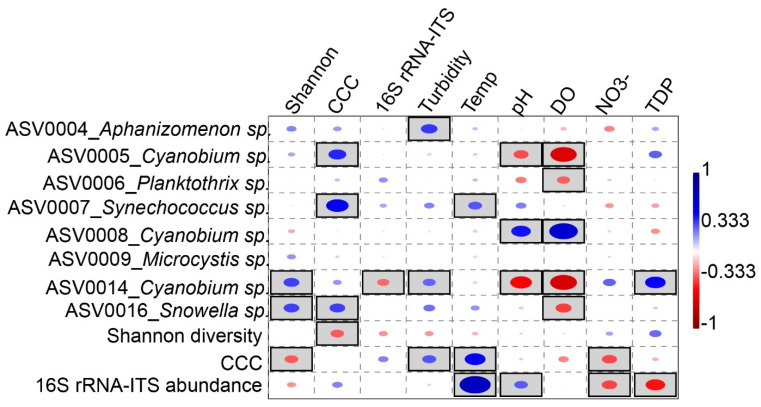
Correlation of pelagic *cyanobacteria* communities with environmental parameters: Rank-based Spearman correlation coefficients of the most abundant *cyanobacteria* ASVs, total *cyanobacteria* composition, and *cyanobacteria* 16S rRNA-ITS gene abundance with internal physicochemical properties in Lake Tiefer See. Gray-shaded squares are significant at *p* < 0.05 with Bonferroni p-value correction. Blue circles show positive, whereas red circles show negative correlations. Shannon diversity = *cyanobacteria* diversity, Temp. = temperature, DO = dissolved oxygen, NO_3_^–^ = nitrate, TDP = total dissolved phosphorus, and 16S rRNA-ITS = 16S rRNA gene internal transcriber spacer (ITS) region of cyanobacteria.

A rank-based correlation analysis between *cyanobacteria* ASVs and a significant subset of lake environmental variables revealed pelagic cyanobacterial abundance measures; *cyanobacteria* composition and 16S rRNA-ITS gene copy numbers to be positively correlated with temperature (*n* = 47, *p* < 0.05, *R_S_* = 0.5, and 0.75, respectively; Figure 6; Tables S2 and S6), and negatively correlated with NO_3_^–^ (*p* < 0.05, *R_S_* = −0.4 and −0.4, respectively). Additionally, 16S rRNA gene copy numbers were negatively correlated with TDP (*p* < 0.05, *R_S_* = 0.5). Among the most abundant taxa, ASV0004 assigned to *Aphanizomenon* and ASV0014 assigned to *Cyanobium* were positively correlated with turbidity (*p* < 0.05, *R_S_* = 0.41, and 0.31, respectively), whereas ASV0014 was the only taxon that correlated positively with TDP (*p* < 0.05, *R_S_* = 0.5). Furthermore, positive correlations with pH (*p* < 0.05, R_S_ = 0.46) and DO (*p* < 0.05, *R_S_* = 0.68) occurred for ASV0008 assigned to *Cyanobium*. In contrast, ASVs 0005 and 0014 also assigned to *Cyanobium* were negatively correlated with pH (*p* < 0.05, R_S_ = −0.4 and −0.51, respectively) and DO (*p* < 0.05, *R_S_* = −0.63, and −0.64, respectively). Additionally, ASVs 0006 and 0016 assigned to *Planktothrix* and *Snowella*, respectively, were negatively correlated with DO (*p* < 0.05, *R_S_* = −0.31 and −0.38, respectively). Among all ASVs, only ASV0007 assigned to *Synechococcus* showed a positive correlation with temperature (*p* < 0.05, *R_S_* = 0.33).

## 4. Discussion

### 4.1. Tracing Cyanobacteria from the Water Column into the Sediment

This study used high throughput sequencing and lake monitoring to trace the deposition of pelagic cyanobacterial taxa in the sediments of Lake Tiefer See. We correlated pelagic cyanobacterial community data with in-lake physicochemical parameters to identify taxa, whose sedimentary records have future research prospects as potential paleolimnological proxies. Our comparative analysis of pelagic and deposited *cyanobacteria* revealed that the deposited community is representative of pelagic communities. This observation adds to previous DNA-based and traditional paleolimnological findings [7,38,39]. However, unlike these previous studies, differences were observed in the relative abundances of individual *cyanobacteria* taxa, such as *Planktothrix* and *Aphanizomenon*, between the water column, sediment trap material, and surface sediments. These differences may be explained by different deposition patterns of these taxa because of ecology, physiology, and spatiotemporal dynamics [40,41], as well as taphonomic processes, such as alteration or deterioration of DNA along the water column during transport to the sediments. 

We further observed that alpha diversity indices (richness and evenness) did not change significantly throughout the water column, and between the sediment traps and surface sediments, indicating that the changes in the relative abundances of individual taxa had no significant effect on the overall *cyanobacteria* species richness and the distribution of single species (evenness) throughout the water column. This supports our finding that sediment-deposited *cyanobacteria* composition is significantly representative of those in the water column and is important for paleolimnological reconstruction of microbial groups from aquatic sediments. Our observations are consistent with a study on the vertical distribution of protistan taxa (at 2 and 130 m water depth) in Lake Bourget, which also revealed that richness (number of phylogenetic units) within the different phyla at both sampled depths were comparable [20]. 

In contrast, the decline in *cyanobacteria* abundance (qPCR) from the meta- to hypolimnion sediment traps and from 10 m water depth downwards (Figure 4b) could be attributed to taphonomic processes, such as decomposition and the effect of zooplankton grazing communities, such as rhizopods, ciliates, rotifers, and crustaceans [42]. A minimum of dissolved oxygen between 11 and 13 m water depth, e.g., for August (Figure S6), points to the formation of a metalimnetic oxygen minimum (MOM) resulting from the heterotrophic decomposition of the deep chlorophyll maximum-forming taxa such as *Planktothrix*, *Cyanobium*, and *Synechococcus*. This is supported by the decrease in cyanobacterial abundance by one order of magnitude between 10 m (1.8 × 10^6^) and 15 m (4.1 × 10^5^) water depths, respectively (Figure 4b). 

Altogether, our data suggest that although taphonomic processes may lead to a decrease in the number of quantifiable *cyanobacteria* at deeper depths, the composition and alpha diversities of deposited cyanobacterial communities are statistically comparable to those of the water column. Additionally, conditions in Lake Tiefer See, such as its hard water with calcite production that facilitates rapid sedimentation in spring and summer [27], bottom water anoxia, and low bottom water temperatures coupled with intact laminated sediments [28], are all likely to promote DNA preservation in the sediments [21,24]. Sediment-deposited *cyanobacteria* in Lake Tiefer See are thus promising candidates for reconstructing pelagic cyanobacterial dynamics of the past [43].

### 4.2. Taxa with High Deposition

Unfavorable limnological conditions, such as a decrease in light intensity and nutrient depletion, especially nitrogen, can result in a breakdown of *Aphanizomenon* summer blooms followed by their sedimentation [44,45], likely explaining their high abundance in sediments (Figure 4a, Figure S3a). Under the aforementioned unfavorable conditions, senescent *Aphanizomenon* cells lose the ability to regulate their own buoyancy, thus sinking or producing akinetes that equally settle in sediments [46]. Furthermore, physiological features, such as higher biomass production, larger cell sizes (e.g., for akinetes 40–220  ×  6–10.8 μm [47]) and cluster formation in *Aphanizomenon* promote their sedimentation compared to non-colonial unicellular and filamentous *cyanobacteria* taxa [48]. The high abundance of *Aphanizomenon* in the sediment traps in fall when the stratification of the water column weakened (Figure S3a), may be because of wind-induced downward transport of senescent cells and akinetes [48,49]. It is unlikely that deposited *Aphanizomenon* either actively (via buoyancy regulation) or passively (via remobilization) reinvaded the water column from surface sediments in fall. This is based on the premise that senescent cells, though still alive, cannot regulate their buoyancy, and any remobilized akinetes will not grow into vegetative cells because of unfavorable conditions (see above) [40,45]. 

The higher abundance of *Snowella* especially in the hypolimnion trap, and sediment relative to the water column could be attributed to their ability to form colonies [50], similar to the aggregate formation of *Aphanizomenon*. Currently, there is sparse literature on *Snowella* sediment deposition and its occurrence in deep temperate lakes. To date, they are known to be common in lakes of varying trophic states in central Europe and the Baltic Sea [51]. A GenBank analysis of the *Snowella* sequences obtained from this study showed that the main ASV was 100% similar to the sequences of the *Snowella litoralis* strain OTU35S07 isolated from the eutrophic Lake Tuusulanjärvi, Finland [50]. However, unlike that in Lake Tuusulanjärvi, where *Snowella* was recorded in samples from the upper waters (0–2 m), we showed a previously unknown bottom water peak in their population and a high sedimentation in the deep hard-water temperate Lake Tiefer See. 

Overwintering communities of *Microcystis* in sediments may explain their high abundance in traps and surface sediments compared to the water column (Figure 4a; Figures S3a and S5b) [52]. *Microcystis* communities in temperate lakes are known to escape unfavorable conditions, such as poor nutrients, lower temperature, and insufficient light in the water column by forming ‘dormant’ colonies (resting stages), which settle on sediments (overwintering). The density gradient in the thermocline during stratification serves as a barrier hindering the migration of *Microcystis* from deeper waters into the euphotic zone [49]. The physiological barrier caused by the gradient could contribute to the very low (<1%) relative abundance of *Microcystis* in the upper water column during its usual bloom season in late summer to fall [53]. This assumption is supported by the fact that thermal stratification in Lake Tiefer See (2019) ended in late November (Figure 2a,b). 

### 4.3. Taxa with Poor Deposition

Considering the all-year presence and abundance of *Planktothrix* in the metalimnion of the water column, their high abundance in sediment traps, especially in the metalimnion trap, and the sediment was expected (Figure 4a). The observed disproportionate abundance between the water column and sediment trap samples is possibly because of *Planktothrix* occurrence as single filaments containing gas vesicles, which hinder their rapid sedimentation [41]. Taphonomic processes, such as heterotrophic decomposition and DNA degradation during transport along the water column, as well as grazing by zooplankton, e.g., Daphnia and Cyclops [54], could be other factors explaining the low abundance of *Planktothrix* in sediments. As discussed earlier, the formation of a temperature gradient (7–13 m water depth; Figure 2a) during lake stratification appears to favor the dominance of *Planktothrix* in the low-light metalimnion (10 m; Figure 4a). In this layer, *Planktothrix* abundance contributes to deep chlorophyll maximum (DCM) formation, as shown by the peak in Chl*a* (Figure 2e; Figure S6). The temperature gradient also likely acts as a physical barrier preventing rapid sedimentation; thus, promoting heterotrophic decomposition of *Planktothrix* in the water column [41]. The observed DO-minimum at 11–13 m water depth suggests that DCM-forming taxa, such as *Planktothrix*, are decomposed at this water depth, leading to the formation of the metalimnetic oxygen minimum (MOM) zone (Figure S4). This is supported by a reduction in the relative abundance of *Planktothrix* between 10 (>40% of all cyanobacteria) and 15 m (<20%) water depths (Figure 4a). Wentzky et al. (2019) equally observed a connection between the increased decomposition of dead organic material resulting from *Planktothrix* and the development of the MOM [55]. The development of the MOM zone indicates that as particulate organic matter sinks from the epilimnion into a zone of lower temperatures and higher water density (metalimnion), particle retention time increases, favoring heterotrophic decomposition in the metalimnion [55]. It is not likely that the *Planktothrix* abundance disparity is caused by methodological limitations, such as primer amplification bias, because the same primer set was used to amplify the *cyanobacteria* 16S rRNA gene in the water and sediment samples. Additionally, complete degradation and/or fragmentation of *Planktothrix* DNA seems unlikely, because Kyle et al. [56] used qPCR assay to amplify and quantify 383 bp fragments of this taxon from up to 50-year-old sediments from the dimictic Lake Gjersjøen (max. depth = 64 m) [57]. Thus, while data from the spatio-temporal integrated sediment traps (~1 month) reveal extremely low abundance of the potential toxin-producing *Planktothrix*, single-point sampling of the water column, however, show they are the most abundant metalimnetic taxa in Lake Tiefer See. This means that relying on sedimentary DNA sequencing based on universal *cyanobacteria* marker alone would be insufficient to reveal previous pelagic *Planktothrix* importance. Therefore, future paleolimnological reconstructions based on sedaDNA may require an additional molecular approach (e.g., via qPCR assay [29,57]) specifically targeting *Planktothrix* to reveal the history of their past pelagic importance in Lake Tiefer See.

Factors, such as rapid DNA degradation because of their small size (<0.2 µm; [58]), grazing by nano-flagellates, e.g., *Ochromonas* sp. [59], and/or the presence of a density gradient, which impedes their sedimentation [60], might explain the low abundance of *Cyanobium* ASV0008 in sediments (Figure 4a, Figure S3a). However, *Cyanobium* ASV0020 with high sediment deposition may be colony-forming in nature. Although studies on the ecology and physiology of colonial *Cyanobium* in freshwater ecosystems are still sparse, most are thought to be part of a metaphyton community associated with littoral and benthic sediments [61]. While our data suggest the identification of possible *Cyanobium* ecotypes based on conserved small subunit ribosomal ribonucleic acid (SSU rRNA) genetic markers, future research should focus on isolating and obtaining full genome sequences that could provide information on the factors influencing the sedimentation of individual *Cyanobium* ecotypes.

### 4.4. Linking Cyanobacteria Community Dynamics with Environmental Data 

Comparison of *cyanobacteria* assemblages from water and sediment matrices revealed that overall *cyanobacteria* composition as well as ASVs assigned to *Synechococcus*, *Aphanizomenon*, and *Snowella* are potential proxies for paleoclimate reconstruction (Figure 6). This is because of their high sediment representation and significant correlation of their pelagic fraction with lake internal physicochemical parameter(s). Their suitability as proxies is based on the assumption that with high sedimentation, coupled with optimal DNA preservation conditions at the time of deposition, e.g., bottom water anoxia, and absence of burrowing organisms [19], their DNA could be reconstructed from long-term sediment records. The overall pelagic *cyanobacteria* composition and that of *Synechococcus* were positively correlated with water temperature. Since the overall sediment-deposited *cyanobacteria* composition is representative of the water column (*p* < 0.05; Table 1), and *Synchococcus* had a high abundance in the sediment matrices (its DNA is also known to be well preserved in sediments [14,62]), coupled with positive correlations to temperature, they could be useful as proxies for temperature in paleolimnological reconstructions. In temperate lakes, temperature has been shown to be a major factor leading to an increase in total *cyanobacteria* and, in particular, *Synechococcus* biomass [63]. Furthermore, reconstructing a taxon in high abundance from late Holocene sediments could point to a bloom and potential toxin production in the water column at the time of deposition [64]. This was illustrated recently in Lake Tiefer See, where previous eutrophication was inferred from the increased abundance of operational taxonomic units assigned to *Aphanizomenon* which positively correlated with increased TN values in sediments [43]. In summary, our study provides information for establishing *cyanobacteria* as proxies for long-term paleoclimate reconstructions. This could be further approached using structural equation modeling (SEM or path analysis) to evaluate directed relationships between dependent and independent variables and not just sheer correlations [65].

## 5. Conclusions

Our study assesses the deposition of freshwater *cyanobacteria* based on high resolution taxonomic profiling of both pelagic and sedimented taxa using DNA-based techniques. It is the first study providing strong arguments for a skewed representation in taxonomic abundances of sediment-deposited *cyanobacteria* composition in a temperate hard-water lake with annual mixing. It reveals two critical findings regarding cyanobacterial sedaDNA reconstructions using universal gene markers. First, taphonomic processes occurring along the water column during sedimentation as well as post-sedimentary processes challenge an approximate estimation of some taxa from sediments, such as those occurring as single filaments (e.g., *Planktothrix*). Second, aggregate- (e.g., *Aphanizomenon*) and colony- (e.g., *Snowella*) forming taxa seem to be less impaired by these taphonomic processes. That is, while factors, such as predation, heterotrophic decomposition, and DNA degradation in the water column, may lead to a poor representation of solitary or single filamentous cells, the ability to form aggregates or colonies in *Aphanizomenon* and *Snowella*, respectively, could be responsible for their overrepresentation in sediments. Our study thus reveals limitations of using high-throughput sequencing analysis based on sediments alone to infer pelagic *cyanobacteria* dynamics. Therefore, applying additional qPCR assay targeting specific taxa may potentially overcome the amplification biases observed in high-throughput sequence-based analyses. This is valuable knowledge for future DNA-based paleolimnological studies, especially in lakes without available long-term monitoring data, because obtaining reliable information about past limnological conditions using *cyanobacteria* sedaDNA as indicators will depend on an accurate estimation of species abundance. This means that complementing pelagic surveys of *cyanobacteria* with in situ sediment traps is crucial for optimally tracking their sediment deposition in lakes. Hereafter, results from such a combined survey will reveal relevant pelagic taxa that may require special targeting during sedaDNA reconstruction. 

## Figures and Tables

**Table 1:** One-way PerMANOVA on cyanobacterial communities grouped into epi-, meta-, and hypolimnion in the water column, meta-, and hypolimnion of the sediment traps and surface sediments. Summary presents overall test statistics. Pairwise analysis shows Bonferroni corrected *p*-values (values in bold are significant at *p* < 0.05) above the diagonal and corresponding *F*-values below. Where ‘W’ indicates water column samples from the epi-, meta- and hypolimnion. The ‘ST’ indicates sediment trap material from the meta- and hypolimnion and ‘Sed’ represents the surface sediment samples.

PERMANOVA		Pairwise	Epi	Meta	Hypo	TrapM	TrapH	Sed
**Summary**		**Epi**		**0.012**	0.0775	**0.0015**	**0.0015**	0.786
Permutation N:	9999	**Meta**	3.962		**0.027**	**0.0015**	**0.0015**	0.8835
Total sum of squares:	8.29	**Hypo**	3.244	4.66		**0.0015**	**0.0015**	1
Within-group sum of squares:	5.438	**TrapM**	8.943	10.24	5.544		1	1
*F*:	6.085	**TrapH**	11.15	12.78	6.638	0.4568		1
*p* (same):	**0.0001**	**Sed**	4.397	5.151	4.244	0.7717	1.116	

## Data Availability

Sequencing data and metadata are deposited in the European Nucleotide Archive (ENA) under BioProject accession number PRJEB40406 and sample accession numbers ERS5083533-ERS5083564 (trap material and surface sediment samples) and ERS5083566-ERS5083644 (water samples).

## Supplementary Materials

The following are available online at https://www.mdpi.com/article/10.3390/microorganisms9081778/s1, Figure S1: Distribution of all *cyanobacteria* ASVs among sample types of Lake Tiefer See, Figure S2: Variation of environmental variables from water and sediment traps of Lake Tiefer See, Figure S3: *Cyanobacteria* distribution and abundance in Lake Tiefer See, Figure S4: Rank-based Spearman correlation, Figure S5: Heatmaps of relative abundances of all amplicon sequence variants (ASVs) assigned to (a) *Aphanizomenon*, (b) *Microcystis*, and (c) *Snowella*, Figure S6: Chlorophyll-a and dissolved oxygen for the month of August 2019 throughout the water column of Lake Tiefer See. Table S1: Statistical analyses of sequencing analysis and alpha diversity data, Table S2: Rank-based Spearman correlation coefficients, Table S3: Similarity percentage (SIMPER) test on the *cyanobacteria* ASVs, Table S4: Total sulfide measurements from water column samples, Table S5: Nitrate measurements from water column samples, Table S6: Results of ANOVA and Tukey’s pairwise statistics, Table S7: Significant explanatory parameters used in the Spearman correlation analysis.
