# Supplementary material for: From Water into Sediment—Tracing Freshwater Cyanobacteria via DNA Analyses
Source: Microorganisms. 2021 Aug 21;9(8):1778. doi: 10.3390/microorganisms9081778 (PMC8400057; doi:10.3390/microorganisms9081778)
Supplement: Supplementary file 1 [file microorganisms-09-01778-s001.zip › microorganisms-1328325-supplementary.pdf]

# Supplementary Material

## From water into sediment - tracing freshwater cyanobacteria via DNA analyses

Ebuka Canisius Nwosu*, Patricia Roeser, Sizhong Yang, Lars Ganzert, Olaf Dellwig, Sylvia Pinkerneil, Achim Brauer, Elke Dittmann, Dirk Wagner and Susanne Liebner

*** Correspondence:**
enwosu@gfz-potsdam.de

**This file includes;**

**Supplementary information:** Extended Materials and Methods

**Supplementary Figures**

Figure S1: Distribution of all cyanobacteria ASVs among sample types of Lake Tiefer See.

Figure S2: Variation of environmental variables from water and sediment traps of Lake Tiefer See.

Figure S3: Cyanobacteria distribution and abundance in Lake Tiefer See.

Figure S4: Rank-based Spearman correlation

Figure S5: Heatmaps of relative abundances of all amplicon sequence variants (ASVs) assigned to a) Aphanizomenon, b) Microcystis, and c) Snowella.

Figure S6: Chlorophyll-a and dissolved oxygen for the month of August 2019 throughout the water column of Lake Tiefer See.

**Supplementary Tables**

Table S1: Statistical analyses of sequencing analysis and alpha diversity data.

Table S2: Rank-based Spearman correlation coefficients.

Table S3: Similarity percentage (SIMPER) test on the cyanobacteria ASVs.

Table S4: Total sulfide measurements from water column samples.

Table S5: Nitrate measurements from water column samples.

Table S7: Significant explanatory parameters used in the Spearman correlation analysis

Supplementary Information: Extended Materials and Methods

Study site. The hard water oligo-mesotrophic Lake Tiefer See (near Klocksin; 53°35.5'N, 12°31.8'E; 62 m a.s.l.) is part of the southern Baltic Lake District and was formed during the final stage of the last glacial period (~13,000 years ago) (Dräger et al., 2017; Fig. 1a, b). Lake Tiefer See is part of a subglacial gully system in a morainic terrain located in Nature Park 'Nossentiner/Schwinzer Heide.' Today, the lake is connected to Lake Hofsee in the south through a shallow sill, whereas the connection to Lake Flacher See in the north has been channelized into a tunnel after the construction of a railway dam between the two lakes (CE 1884–1886) and is currently largely interrupted. Lake Tiefer See has a surface area of approximately 0.75 km2, and the catchment area of approximately 5.5 km2 is dominated by glacial till. Although the catchment is mainly used for agriculture (Theuerkauf et al. 2015), the direct shore-line of the lake is covered by a fringe of trees, and there is no anthropogenic infrastructure, such as buildings or roads at the lakeshore. The lake has a maximum depth of 62 m and no major inflows or outflow. The annual lake mixing is either mono- or dimictic, depending on the formation of a winter ice cover (Kienel et al. 2017). The study site is characterized by a warm-temperate climate during the transition from oceanic to continental conditions. Mean monthly temperatures range from 0°C in January to 17–18°C in July, with maxima up to 30°C and minima down to -5°C. Mean monthly precipitation varies between ~40 mm during winter and ~60 mm in summer, with a mean annual precipitation of 560–570 mm (Kienel et al. 2017). Lake Tiefer See has been the focus of an extensive and high-resolution climate monitoring program since the beginning of the last decade (Roeser et al. 2021). The lake is well-preserved and seasonally laminated sediments have aided climate reconstructions spanning the last 6,000 years (e.g., Dräger et al. 2019).

Collection of water, sediment trap material, and sediment cores

Water samples were collected monthly between January 30 and November 28, 2019, at the floating weather monitoring station, located at the maximum depth of the lake (62 m; Fig. 1b). The sampled water depths were 1, 3, 5, 7, 10, 15, 20, 40, 45, and 50 m. A sample volume of 250 mL of lake water was collected for the upper 1, 3, and 5 m water depths and pooled (750 mL), representing the epilimnion. Likewise, 375 mL volumes were collected for the lower 45 and 50 m water depths and pooled (750 mL), representing the bottom waters. For each of the other 7, 10, 15, 20, and 40 m water depths, 750-mL water samples were collected. The samples were collected in sterile glass bottles (Schott Duran®, Germany), filtered within 24 h after fieldwork using 0.2 µm cellulose filters (Sartorius AG, Germany), and the filters were stored at -20°C until nucleic acid extraction.

The suspended particulate matter was trapped with four-cylinder traps (KC Denmark A/S total active area 0.0163 m2 for the four cylinders) at two depths in the water column (Fig. 1c). One trap was anchored in the metalimnion (12 m water depth), whereas the second trap was secured in the hypolimnion (55 m water depth). For both depths, the traps were emptied monthly between April and November 2019, i.e., the annual interval of increased lake productivity. The trapped material was transferred into 2 L plastic bottles, which were allowed to settle overnight at 4°C. An equal volume of water was discarded from the bottles, and the remaining suspension was transferred into sterile 50 mL Falcon tubes (Fischer Scientific GmbH, Germany). The tubes containing suspended particulate matter were then centrifuged at 4000 g for 5 min. Subsequently, the supernatant was discarded, and the pellet was stored in Falcon tubes at -20°C until nucleic acid extraction.

A surface sediment core (TSK19-SC6; 115 cm length) was collected on August 29, 2019, from the point of maximum water depth (62 m) using a 90 mm UWITEC piston corer. The temperature of the upright positioned core was maintained at 4 °C during transportation and short-term storage at the facilities of the GFZ Potsdam. To preserve the uppermost varves, the core was allowed to dry in a vertical position at 4 °C for approximately 2 weeks before longitudinal splitting. After splitting the core, one half was lithologically described. A triplicate 0–2 cm subsample of the other core half was stored in sterile 15 mL Falcon tubes at -20°C until nucleic acid extraction. All core handling was performed under clean

conditions in a room where no molecular biological work had been previously conducted to avoid contamination.

Lake physicochemical properties

Measurements of the physicochemical parameters, such as temperature, pH, dissolved oxygen (DO), turbidity, and chlorophyll-a (Chla) in the water column were conducted automatically using a multi-parameter water quality probe (YSI 6600 V2, Yellow Springs USA), in 1-m steps and 12 h resolution. Owing to technical problems, in February, data were only collected on 2 d (1st and 28th), and in March, no data were recorded between the 2nd and 10th. Water temperature was additionally measured using 26 stationary data loggers (HOBO Water Temp Pro v2, Onset USA) in 1-m steps from 0 to 15 m and 5-m steps from 15 to 55 m water depth (Fig. 1c). Water samples for nitrate (NO3–) and total dissolved phosphorus (TDP) analyses were also collected monthly, in parallel with those for molecular analysis. Water samples for total sulfide were collected from the hypolimnion in October and November. Water samples for nitrate measurements were analyzed at the German Research Centre for Geosciences (GFZ), whereas TDP and total sulfide were conducted at the Leibniz Institute for Baltic Sea Research Warnemünde (IOW). Nitrate was measured by suppressed ion chromatography using a SeQuant SAMS anion IC suppressor (EMD Millipore, Billerica, Massachusetts), an S5200 sample injector, a 3.0 × 250 mm LCA 14 column, and an S3115 conductivity detector (all Sykam, Fürstenfeldbruck, Germany). The eluent was 5 mM Na2CO3, 20 mg L-1 4–hydroxybenzonitrile and 0.2% methanol. The flow rate was set to 1 mL min-1, and the column oven temperature was to 50°C. The detection and quantification limits were calculated based on signal-to-noise (S/N) ratios of 3 and 10, respectively. All samples were measured in triplicate, and every 10 injections a standard was measured to check for drift. Reproducibility was always better than 5%, and the detection limit ranged between 1–4 μM. For TDP, the water samples were immediately filtered using 0.45 μm syringe filters and acidified with sub-boiled HNO3 to 2 vol%. The TDP was measured by inductively coupled plasma optical emission spectrometry (ICP-OES; iCAP 7400, Duo, Thermo Fisher Scientific) using external calibration and Sc as the internal

standard. Precision and trueness were checked with the international reference material SLRS-6 (NRCC) spiked with 20 μg L-1 P, which were 9.6% and 5.7%, respectively. After fixation of 50 mL of an aliquot of the filtered water sample with 500 μL 20 vol% zinc acetate solution, total sulfide was determined spectrophotometrically according to the Cline method (Cline, 1969) with a precision of 3.2% (Dellwig et al. 2019).

The sediment fluxes (sediment flux: g m-2 d-1) were determined from the weighted and freeze-dried trapped sediment material. The samples were homogenized using an agate mortar. Prior to total organic carbon (TOC) and total nitrogen (TN) determination, replicate aliquots (0.2 mg) were decalcified in situ in Ag capsules (20% HCl and dried at 75°C). The TOC and TN contents were measured using the elemental analyzers NC2500 and EA3000-CHS Eurovectors, respectively, and were used to calculate the atomic C:N ratio. CaCO3 content was estimated after obtaining the total inorganic carbon content (TIC = TC–TOC) and multiplying by a factor of 8.33, which is the percentage of molecular weight in inorganic carbon in the calcium carbonate structure.

Molecular analyses

All nucleic acid extractions were conducted in a clean laboratory where no polymerase chain reaction (PCR) was performed prior to the extraction, following established methods and precautions to limit contamination. Water sample DNA extraction was performed at different times than the sediment samples to avoid cross-contamination. Additionally, the sediment traps were processed independently from the surface sediment cores. Because of water column turnover in January and February, water samples for molecular analyses from 1, 3, 5, 7, and 10 m water depths were pooled and reported as the mean depth (5 m). Water depths of 40, 45, and 50 m were equally pooled and reported as the mean depth (45 m). In March, when temperatures began to increase, four depths in the water column were reported as follows: 5 (1, 3, 5, and 7 m pooled), 10, 20, and 45 m (40, 45, and 50 m pooled). Genomic DNA (gDNA) of water samples was extracted from the filters using the DNeasy PowerWater Kit (QIAGEN, Hilden, Germany) following the manufacturer's specifications. The gDNA from the

homogenized sediment trap samples and surface sediment samples was extracted from approximately 0.75 g using the DNeasy PowerSoil Kit (QIAGEN, Hilden, Germany). The extracted gDNA from all samples was eluted in 100 µL elution buffer and stored at -20°C until downstream analysis. Cyanobacteria quantification Total cyanobacteria were quantified with a SYBR Green qPCR assay that specifically amplified the cyanobacterial 16S rRNA-ITS (internal transcribed spacer) region using the primers CSIF (5'-GYCACGCCCGAAGTCRTTAC-3') and 373R (5'-CTAACCACCTGAGCTAAT-3') (Janse et al., 2003). The final volume of the qPCR reactions was 20 µL, which consisted 10 µL of KAPA HiFi 2x SensiFAST SYBR® FAST qPCR Master Mix (Sigma-Aldrich, Germany), 0.2 µL of each forward and reverse primer (100 µM, Biomers), 5.8 µL PCR water, and 4 µL of template DNA. The qPCR program consisted of an initial polymerase activation step (95°C for 15 min), followed by 40 cycles of denaturation at 94°C for 60 s, annealing at 60°C for 60 s, and extension at 72°C for 60 s. The qPCR program was followed by a melting curve step from 70°C to 95°C at a transition rate of 1°C per 5 s to determine the specificity of the amplification. The amplified products were confirmed using agarose gel electrophoresis. Tenfold dilution standards were prepared from gDNA of Synechocystis for each qPCR assay, which ranged from 4.7 × 107 to 4.7 × 103 copies ng-1 DNA, and was used to create the standard curves from which the quantification cycle (Cq) values were determined (Bustin et al. 2009). The copy numbers of the 16S rRNA-ITS region were calculated following Savichtcheva et al. (2011), and were the mean of triplicates of each sample expressed as cyanobacterial abundance normalized to extracted DNA (copies ng-1 DNA) with a minimum quantification efficiency of 91%. All qPCR assays were performed in triplicate on a CFX96 real-time thermal cycler (Bio-Rad Laboratories, Inc., USA).

Sequence library preparation All consumables and pipets used for PCR were sterilized in a laminar flow hood under UVC light for at least 15 min prior to use. PCR for the Illumina high-throughput sequencing (HTS) libraries was conducted using the cyanobacteria-specific primers CYA359F (5'-CGGACGGGTGAGTAACGCGTG-3') and CYA784R (5'-ACTACWGGGGTATCTAATCCC-3') (Nübel et al. 1997). They amplify a >400-nt-long fragment of the V3–V4 regions of the 16S rRNA gene. The

primers had unique tags (Supplemental Information SI 1) that served to differentiate the samples. The water and sediment samples, as well as the negative control (that is, a reaction with PCR water as a template) were amplified in a PCR reaction of 50 μL, containing 10x Pol Buffer C (Roboklon GmbH, Berlin, Germany), 25 mM MgCl2, 0.2 mM deoxynucleoside triphosphate (dNTP) mix (ThermoFisher Scientific), 0.5 mM each primer (TIB Molbiol, Berlin, Germany) and 1.25 U of Optitaq Polymerase (Roboklon). The volume of the template DNA used in each reaction varied between 1 and 4 μL, depending on the sedDNA concentration. The PCR program included an initial denaturation step at 95°C for 10 min, followed by 35 cycles at 95°C for 15 s, annealing at 60°C for 30 s, extension at 72°C for 45 s, and a final extension step at 72°C for 5 min. To avoid cross-contamination, the PCR reactions were performed monthly after each sampling, and the water samples were processed separately from the sediment samples. The tagged PCR products were then purified with the Agencourt AMPure XP kit (Beckman Coulter, Brea, CA, USA) and eluted in 30 μL DNA/RNA-free water. The purified product was quantified using a Qubit (2.0) fluorometer. Equimolar concentrations of all samples, including two negative purified PCR controls, were pooled into two multiplex libraries (n = 160 samples, including 78 samples and 2 controls per library). The two libraries contained technical replicates of the water samples and biological replicates of the sediment samples. The final libraries were paired-end sequenced (2 × 300 bp) on an Illumina MiSeq system (Eurofins Scientific (Constance, Germany)).

Bioinformatics. The obtained 14,649,824 sequence reads were quality checked on a raw FASTQ file with FastQC (Andrews et al. 2015). The reads were then demultiplexed using the make.contigs function in Mothur (v.1.39.5: pdiff=2, bdiff=1, and default settings for others) (Schloss et al. 2009). Based on the report files, the sequence identifiers were retrieved for those sequences with minimum overlap (length > 25), maximum mismatches (< 5), and the maximum number of ambiguous bases of zero (which means there was no base marked with N'). According to the sequence identifiers from the previous step, the sequences were then extracted with the filterbyname.sh function from BBtools (Bushnell et al. 2017)

from the raw paired-end fastq files. This step was followed by checking and correcting the sequence orientation and removing sample unique barcodes using extract_barcodes.py in QIIME1 (Caporaso et al., 2010). Finally, primers were removed using Cutadapt (Martin 2014). The sequences were then fed to DADA2 (Callahan et al. 2016) for filtering, dereplication, chimera check, sequence merge, and amplicon sequence variant (ASV) calling. Then, QIIME2 (Bolyen et al. 2019) was used for taxonomic assignment against the SILVA138 database (Quast et al. 2013).

Data availability. Sequencing data and metadata are deposited in the European Nucleotide Archive (ENA) under BioProject accession number PRJEB40406 and sample accession numbers ERS5083533-ERS5083564 (trap material and surface sediment samples) and ERS5083566-ERS5083644 (water samples).

Sequence data processing. The 152 samples resulted in a total of 7,297,946 denoised and error-corrected sequences that DADA2 inferred in 2,538 ASVs. We filtered out all ASVs not assigned to cyanobacteria and chloroplasts, and another 43 ASVs that occurred in fewer than three samples over the entire dataset to reduce bias from very rare taxa. The four negative controls that were included in the sequencing run each comprised less than 1% of the reads compared with the sample average and were therefore removed.

## References of the extended materials and methods

Andrews, S., F. Krueger, A. Seconds-Pichon, F. Biggins, and S. Wingett. 2015. FastQC. A quality control tool for high-throughput sequence data. Babraham Bioinformatics. Babraham Inst.

Bushnell, B., J. Rood, and E. Singer. 2017. BBTools software package. PLOS ONE **12**: e0185056, doi:10.1371/journal.pone.0185056

Bustin, S. A., and others 2009. The MIQE guidelines: Minimum information for publication of quantitative real-time PCR experiments. Clin. Chem. **55**: 611-622. doi:10.1373/clinchem.2008.112797

Callahan, B. J., P. J. McMurdie, M. J. Rosen, A. W. Han, A. J. A. Johnson, and S. P. Holmes. 2016. DADA2: High-resolution sample inference from Illumina amplicon data. Nat. Methods. **13**: 581-583. doi:10.1038/nmeth.3869

Caporaso, J. G., and others. 2010. QIIME allows analysis of high-throughput community sequencing data. Nat. Methods. **7**: 335-336. doi:10.1038/nmeth.f.303

Cline, J. D. 1969. Spectrophotometric determination of hydrogen sulfide in natural waters. Limnol. Oceanogr. **14**: 454-458. doi:10.4319/lo.1969.14.3.0454

Czymzik, M., R. Muscheler, F. Adolphi, and others. 2018. Synchronizing 10Be in two varved lake sediment records to IntCal13 14C during three grand solar minima. Clim. Past. **14:** 687-696. doi:10.5194/cp-14-687-2018

Dellwig, O., A. Wegwerth, B. Schnetger, H. Schulz, and H. W. Arz. 2019. Dissimilar behaviors of the geochemical twins W and Mo in hypoxic-euxinic marine basins. Earth Sci. Rev. **193**: 1-23. doi:10.1016/j.earscirev.2019.03.017

Dräger, N., B. Plessen, U. Kienel, M. Słowiński, A. Ramisch, R. Tjallingii, S. Pinkerneil, and A. Brauer. 2019. Hypolimnetic oxygen conditions influence varve preservation and δ13C of sediment organic matter in Lake Tiefer See, NE Germany. J. Paleolimnol. **62**: 181-194. doi:10.1007/s10933-019-00084-2

Dräger, N., M. Theuerkauf, K. Szeroczyńska, S. Wulf, R. Tjallingii, B. Plessen, U. Kienel, and A. Brauer. 2017. Varve microfacies and varve preservation record of climate change and human impact for the last 6000 years at Lake Tiefer See (NE Germany). Holocene **27**: 450-464. doi:10.1177/0959683616660173

Janse, I., M. Meima, W. E. A. Kardinaal, and G. Zwart. 2003. High-resolution differentiation of cyanobacteria by using rRNA-internal transcribed spacer denaturing gradient gel electrophoresis. Appl. Environ. Microbiol. **69**: 6634-6643. doi:10.1128/AEM.69.11.6634-6643.2003

Kienel, U., G. Kirillin, B. Brademann, B. Plessen, R. Lampe, and A. Brauer. 2017. Effects of spring warming and mixing duration on diatom deposition in deep Tiefer See, NE Germany. J. Paleolimnol. **57**: 37-49. doi:10.1007/s10933-016-9925-z

Martin, M. 2014. Cutadapt removes adapter sequences from high-throughput sequencing reads. EMBnet. J. **17**: 10-12. doi:10.14806/ej.17.1.200

Nübel, U., F. Garcia-Pichel, and G. Muyzer. 1997. PCR primers to amplify 16S rRNA genes from cyanobacteria. Appl. Environ. Microbiol. **63**: 3327-3332

Quast, C., E. Pruesse, P. Yilmaz, J. Gerken, T. Schweer, P. Yarza, J. Peplies, and F. O. Glöckner. 2013. The SILVA ribosomal RNA gene database project: Improved data processing and web-based tools. Nucleic Acids Res. **41**: D590-D596. doi:10.1093/nar/gks1219

Roeser, P., N. Dräger, D. Brykała, and others. 2021. Advances in understanding calcite varve formation: New insights from a dual lake monitoring approach in the southern Baltic lowlands. Boreas 50: 419-440. doi:10.1111/bor.12506

Savichtcheva, O., D. Debroas, R. Kurmayer, C. Villar, J. P. Jenny, F. Arnaud, M. E. Perga, and I. Domaizon. 2011. Quantitative PCR enumeration of total/toxic *Planktothrix rubescens* and total cyanobacteria in preserved DNA isolated from lake sediments. Appl. Environ. Microbiol. **77**: 8744-8753. doi:10.1128/AEM.06106-11

Savichtcheva, O., D. Debroas, M. E. Perga, and others. 2015. Effects of nutrients and warming on Planktothrix dynamics and diversity: A palaeolimnological view based on sedimentary DNA and RNA. Freshw. Biol. **60**: 31-49. doi:10.1111/fwb.12465

Schloss, P. D., and others. 2009. Introducing Mothur: Open-source, platform-independent, community-supported software for describing and comparing microbial communities. Appl. Environ. Microbiol. **75**: 7537-7541. doi:10.1128/AEM.01541-09

Theuerkauf, M., N. Dräger, U. Kienel, A. Kuparinen, and A. Brauer. 2015. Effects of changes in land management practices on pollen productivity of open vegetation during the last century derived from varved lake sediments. Holocene. **25**: 733-744. doi:10.1177/0959683614567881

## Supplementary Figures

Figure S1. Distribution of all cyanobacteria ASVs among sample types of Lake Tiefer See

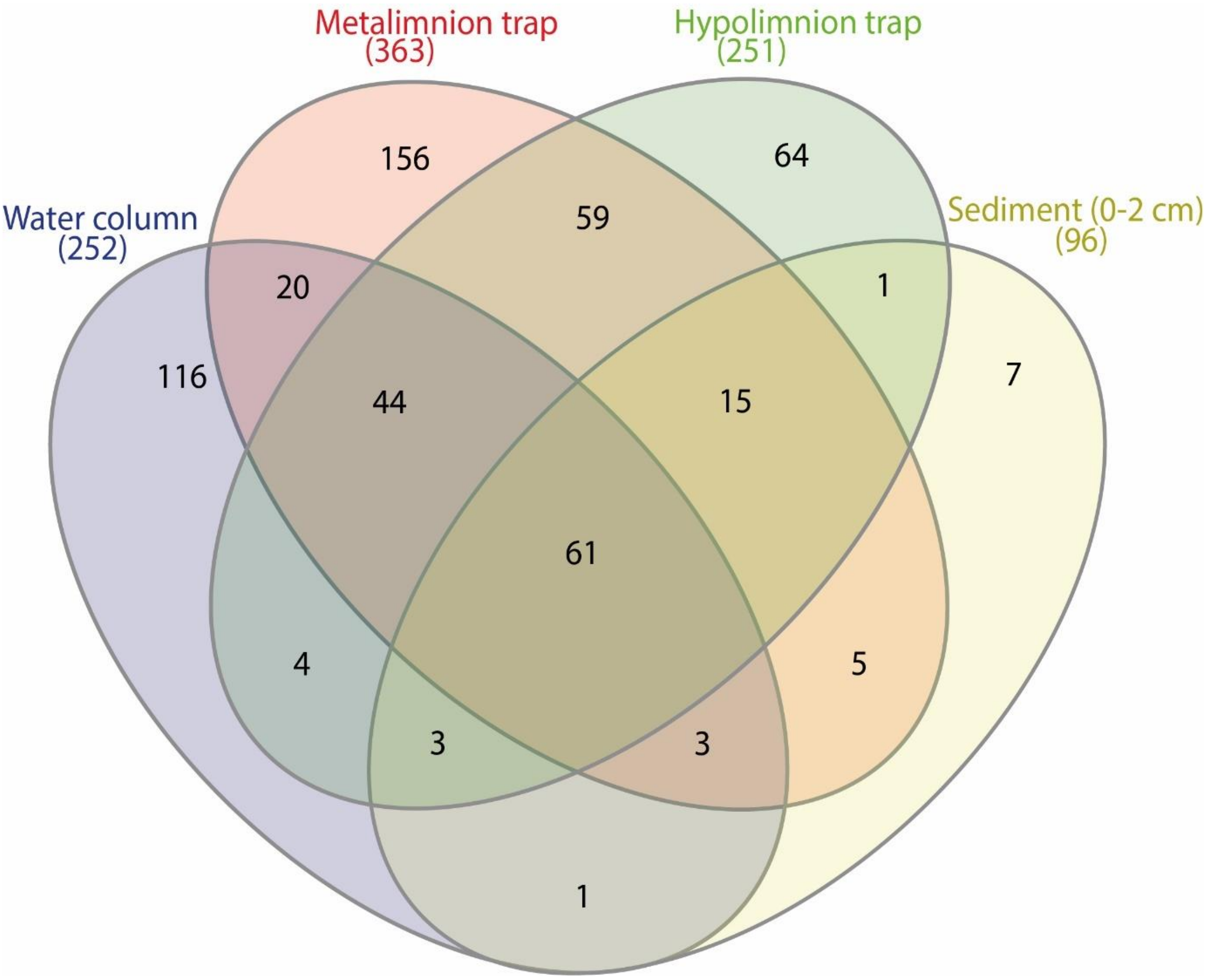

Venn diagram shows distribution of the 559 cyanobacterial amplicon sequence variants (ASVs) among the sample types.

Figure S2. Variation of environmental variables from water and sediment traps of Lake Tiefer See: Principal components analyses (PCA) of environmental data based on Euclidean distance from (a) water column with percentage variation between samples from the thermal stratification zones indicated on the principal components PC1 and PC2 axes. Total variance explained by both axes = 64.14%. Epilimnion n = 19, metalimnion n = 16 and hypolimnion n = 12. (b) meta- (orange; n = 8) and hypolimnion (cyan; n = 8) sediment trap samples. Both PC1 and PC2 together explain 76.61% of total variance among the samples. In both plots the environmental variables are projected as green vectors.

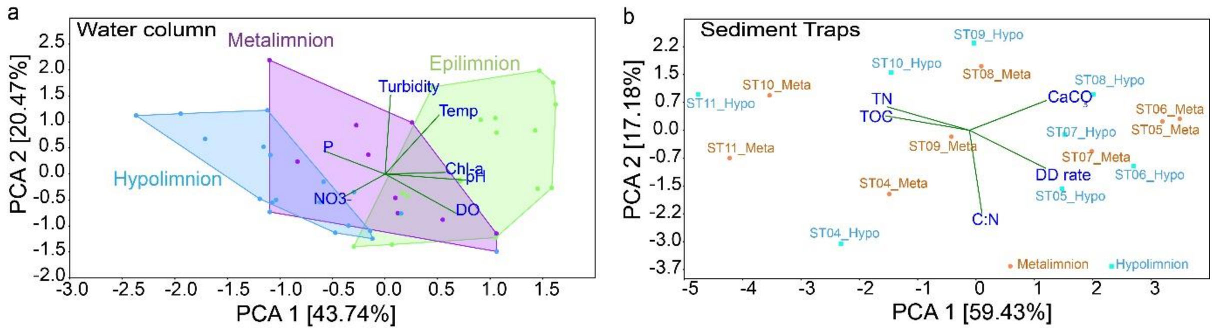


Figure S3. Cyanobacteria community distribution in Lake Tiefer See (a) Bubble plot showing spatial (depth) and temporal (monthly) variation of the most abundant cyanobacteria ASVs (> 1% reads) in 2019, and (b) Cyanobacterial abundance quantified via qPCR with primers that

amplify part of the 16S rRNA internal transcriber spacer (ITS) region of cyanobacteria (data were normalized to ng DNA extracted). Blue vertical lines represent the average abundance in each depth while broken blue vertical line represents overall averages in water column and sediment traps, respectively.

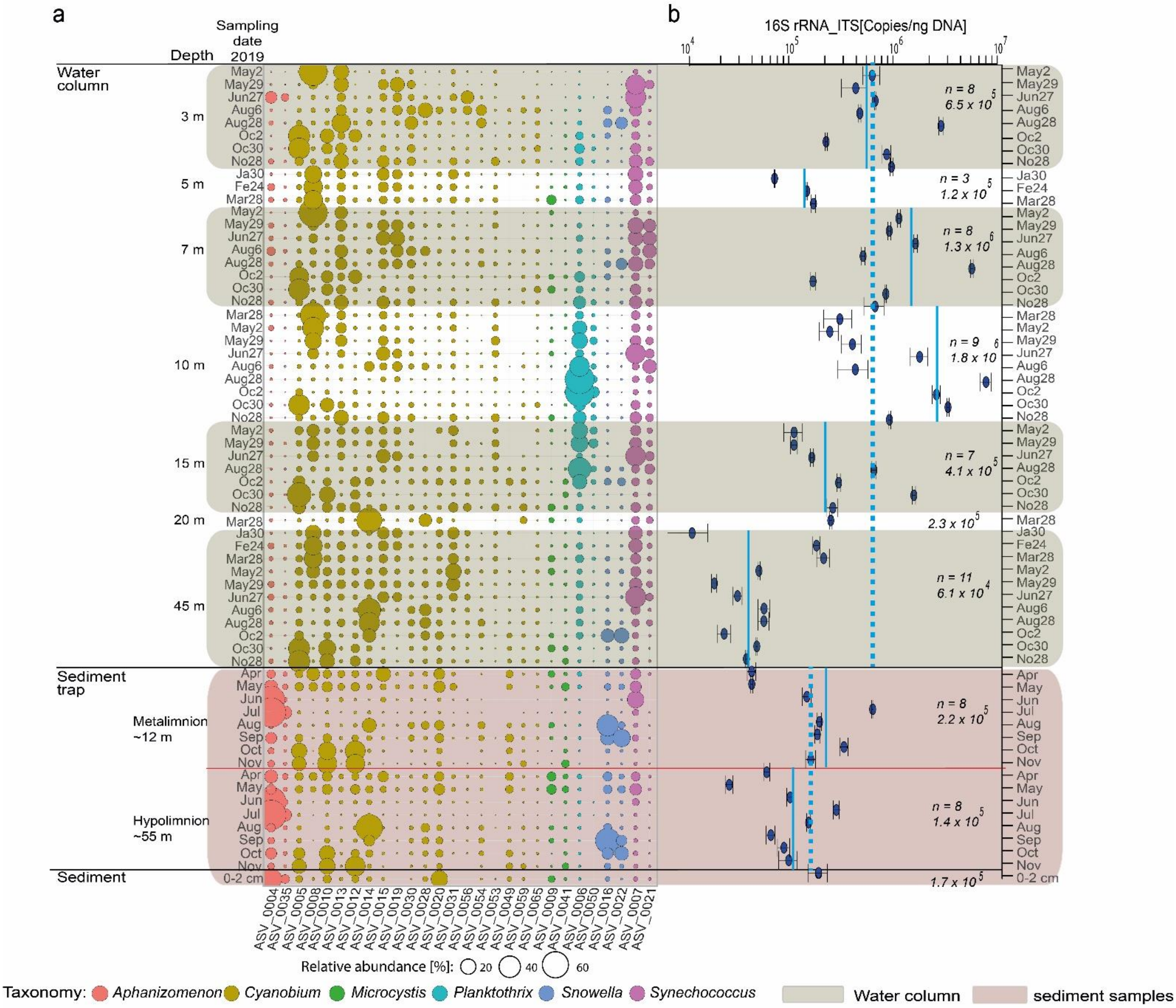


Figure S4 Rank based spearman correlation of the total cyanobacteria community composition from the hypolimnion trap with internal physicochemical parameters in Lake Tiefer See. Blue bubbles show positive while red bubbles show negative correlation. The p values are shown below the figure.

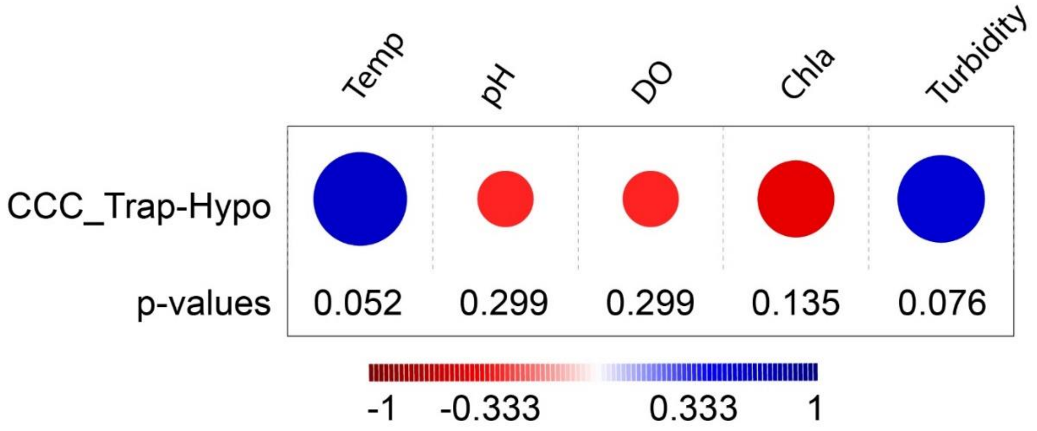


Figure S5 Heatmaps of relative abundances of all amplicon sequence variants (ASVs) assigned to (a) *Aphanizomenon*, (b) *Microcystis*, and (c) *Snowella*. Data is interpolated for 15 days and 5 m water depth. Black circles and red squares represent the depths and timing of sampling of water and sediment traps, respectively.

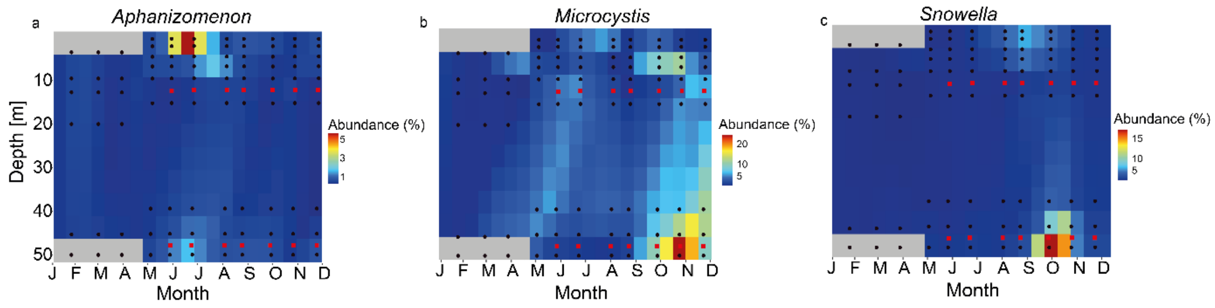


Figure S6. Chlorophyll a and dissolved oxygen for the month of August 2019 throughout the water column of Lake Tiefer See showing the formation of deep chlorophyll maximum (DCM) and the metalimnetic oxygen minimum (MOM) layers in the metalimnion.

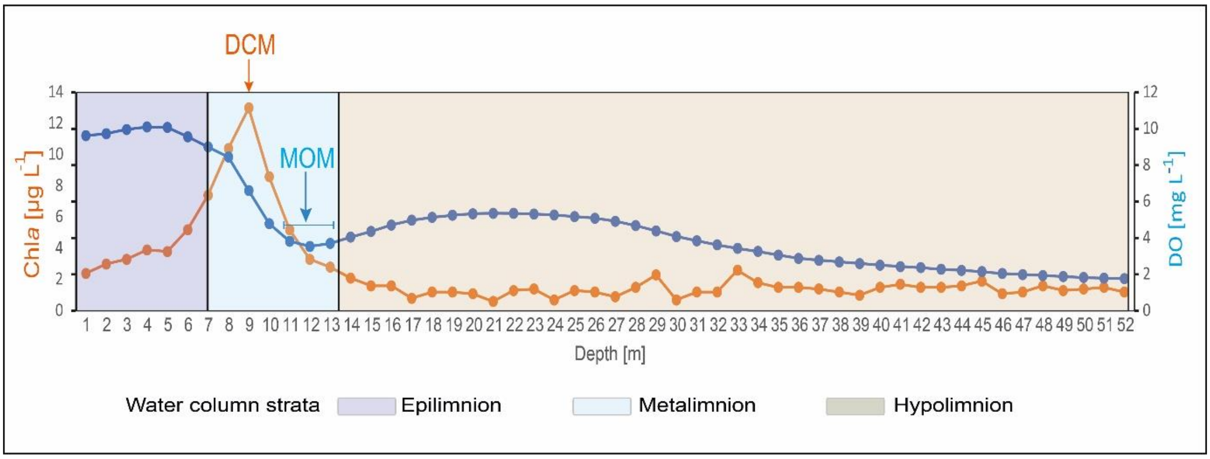


## Supplementary Tables

**Table S1.** Statistical analyses of sequencing analysis and alpha diversity data.

| Sample Site | Sample ID | Raw read counts | Quality filtered read counts | Cyanobacteria read counts | Observed ASVs >1% | Shannon_H >1% | Pielou's Evenness >1% | Barcode sequence-F | Barcode sequence-R |
|---|---|---|---|---|---|---|---|---|---|
| Epilimnion water column | May2_3 | 175384 | 98630 | 81793 | 25 | 1,055 | 0,1149 | CATCCAGT | CGTGATAC |
| | May29_3 | 222133 | 92445 | 72541 | 23 | 1,872 | 0,2827 | CATCCAGT | CTTCTTCC |
| | Jun_3 | 149317 | 48309 | 45708 | 25 | 2,179 | 0,3534 | CATCCAGT | TATCCTCC |
| | Aug6_3 | 150922 | 45764 | 42420 | 26 | 2,648 | 0,5435 | CATCCAGT | AACACCGT |
| | Aug28_3 | 91961 | 31111 | 28751 | 26 | 2,378 | 0,4149 | CATCCAGT | TTACCGCT |
| | Oc2_3 | 281533 | 114661 | 94889 | 26 | 2 | 0,2841 | CATCCAGT | TGAGATGC |
| | Oc30_3 | 62337 | 26642 | 24724 | 27 | 2,139 | 0,3146 | CATCCAGT | GTGCAACT |
| | No_3 | 143307 | 43009 | 33877 | 27 | 2,619 | 0,5081 | CATCCAGT | TGAGCCTA |
| | Ja5 | 165673 | 84153 | 26826 | 27 | 2,307 | 0,3722 | CAACCTCA | CGTGATAC |
| | Fe5 | 141976 | 66220 | 9396 | 25 | 2,38 | 0,4322 | CAACCTCA | CTTCTTCC |
| | Mar_5 | 188223 | 116484 | 6880 | 25 | 2,594 | 0,5353 | CAACCTCA | TATCCTCC |
| | May2_7 | 143438 | 76718 | 53025 | 26 | 1,21 | 0,1289 | CAACCTCA | AACACCGT |
| | May29_7 | 87050 | 32700 | 19614 | 26 | 2,5 | 0,4684 | CAACCTCA | TTACCGCT |
| | Jun_7 | 121635 | 45534 | 28964 | 26 | 2,284 | 0,3776 | CAACCTCA | TGAGATGC |
| | Aug6_7 | 113860 | 41923 | 29158 | 27 | 2,694 | 0,5476 | CAACCTCA | GTGCAACT |
| | Aug28_7 | 125613 | 38114 | 27369 | 27 | 2,685 | 0,5431 | CAACCTCA | TGAGCCTA |
| | Oc2_7 | 165231 | 74945 | 42161 | 27 | 2,288 | 0,3652 | CAACCTCA | ATGGAGGT |
| | Oc30_7 | 110619 | 44863 | 33462 | 27 | 2,169 | 0,3242 | CAACCTCA | CTGAGTCT |
| | No_7 | 98597 | 30472 | 19070 | 26 | 2,605 | 0,5206 | CAACCTCA | GAGGTGAA |
| Metalimnion water column | Mar_10 | 179609 | 90856 | 16944 | 25 | 1,761 | 0,2326 | TGCTTGTC | CGTGATAC |
| | May2_10 | 130479 | 63282 | 12305 | 24 | 2,101 | 0,3405 | TGCTTGTC | CTTCTTCC |
| | May29_10 | 156049 | 63943 | 38722 | 25 | 2,231 | 0,3723 | TGCTTGTC | TATCCTCC |
| | Jun_10 | 142750 | 63870 | 32767 | 26 | 2,236 | 0,3598 | TGCTTGTC | AACACCGT |
| | Aug6_10 | 123174 | 47865 | 32423 | 27 | 2,308 | 0,3723 | TGCTTGTC | TTACCGCT |
| | Aug28_10 | 137861 | 66836 | 50276 | 26 | 1,21 | 0,129 | TGCTTGTC | TGAGATGC |
| | Oc2_10 | 120789 | 70191 | 60084 | 27 | 1,149 | 0,1169 | TGCTTGTC | GTGCAACT |
| | Oc30_10 | 133799 | 49913 | 37460 | 27 | 2,126 | 0,3105 | TGCTTGTC | TGAGCCTA |
| | No_10 | 142473 | 51925 | 30701 | 27 | 2,597 | 0,497 | TGCTTGTC | ATGGAGGT |
| | May2_15 | 81787 | 46493 | 2269 | 20 | 2,568 | 0,652 | TCGTCGCTCG | CTTCTTCC |
| | May29_15 | 80368 | 40667 | 4737 | 22 | 2,47 | 0,5374 | TCGTCGCTCG | TATCCTCC |
| | Jun_15 | 41918 | 18060 | 16238 | 24 | 2,209 | 0,3795 | TCGTCGCTCG | AACACCGT |
| | Aug28_15 | 42116 | 18868 | 17440 | 27 | 2,311 | 0,3735 | TCGTCGCTCG | TGAGATGC |
| | Oc2_15 | 51271 | 20196 | 11927 | 27 | 2,852 | 0,6417 | TCGTCGCTCG | GTGCAACT |
| | Oc30_15 | 50931 | 24740 | 21906 | 27 | 1,738 | 0,2105 | TCGTCGCTCG | TGAGCCTA |
| | No_15 | 48673 | 22522 | 14514 | 27 | 2,884 | 0,6626 | TCGTCGCTCG | ATGGAGGT |
| Hypolimnion Water column | Mar_20 | 95758 | 46951 | 6179 | 22 | 2,047 | 0,352 | TCGTCGCTCG | CGTGATAC |
| | Ja45 | 171550 | 91611 | 34366 | 26 | 2,457 | 0,4487 | AGACGCACTC | CGTGATAC |
| | Fe45 | 114796 | 48626 | 9228 | 25 | 2,476 | 0,4756 | AGACGCACTC | CTTCTTCC |
| | Mar_45 | 221091 | 101049 | 10111 | 26 | 2,639 | 0,5384 | AGACGCACTC | TATCCTCC |
| | May2_45 | 153557 | 88720 | 13370 | 27 | 2,708 | 0,5553 | AGACGCACTC | AACACCGT |
| | May29_45 | 156154 | 95642 | 15194 | 27 | 2,841 | 0,6345 | AGACGCACTC | TTACCGCT |
| | Jun_45 | 177970 | 45534 | 37373 | 27 | 2,426 | 0,4188 | AGACGCACTC | TGAGATGC |
| | Aug6_45 | 80692 | 36232 | 19745 | 27 | 2,084 | 0,2975 | AGACGCACTC | GTGCAACT |
| | Aug28_45 | 129908 | 52656 | 39619 | 27 | 2,439 | 0,4245 | AGACGCACTC | TGAGCCTA |
| | Oc2_45 | 202697 | 110256 | 56113 | 27 | 2,625 | 0,5114 | AGACGCACTC | ATGGAGGT |
| | Oc30_45 | 109925 | 58519 | 42235 | 27 | 1,985 | 0,2696 | AGACGCACTC | CTGAGTCT |
| | No_45 | 116083 | 58165 | 29652 | 27 | 2,126 | 0,3103 | AGACGCACTC | GAGGTGAA |
| Metalimnion Sediment Trap | ST04M | 148116 | 96606 | 25447 | 24 | 2,669 | 0,6011 | GGTAGTTCT | CTGAGTCT |
| | ST05M | 103171 | 76608 | 6266 | 21 | 2,754 | 0,7477 | GGTAGTTCT | GAGGTGAA |
| | ST06M | 103612 | 62935 | 36315 | 21 | 1,095 | 0,1423 | GGTAGTTCT | GGCATGTA |
| | ST07M | 71344 | 54145 | 52049 | 24 | 0,9098 | 0,1035 | CGTAAGTC | CTGAGTCT |
| | ST08M | 87695 | 41455 | 22985 | 25 | 2,15 | 0,3435 | CGTAAGTC | GAGGTGAA |
| | ST09M | 121584 | 65137 | 52867 | 26 | 2,444 | 0,443 | CGTAAGTC | GGCATGTA |
| | ST10M | 83235 | 39122 | 29311 | 22 | 1,779 | 0,2692 | AACTGTCC | GAGGTGAA |
| | ST11M | 394735 | 157394 | 107948 | 26 | 1,768 | 0,2253 | AACTGTCC | GGCATGTA |
| Hypolimnion Sediment Trap | ST04H | 131181 | 104465 | 8796 | 25 | 2,868 | 0,704 | TCACGTACTA | GAGGTGAA |
| | ST05H | 117673 | 85782 | 16330 | 25 | 2,871 | 0,706 | TCACGTACTA | GGCATGTA |
| | ST06H | 70692 | 47525 | 26943 | 26 | 1,062 | 0,1112 | CGCATAGA | GTGCAACT |
| | ST07H | 141180 | 101235 | 76740 | 27 | 0,8603 | 0,08755 | CGCATAGA | TGAGCCTA |
| | ST08H | 62645 | 35007 | 25681 | 25 | 1,648 | 0,2079 | CGCATAGA | ATGGAGGT |
| | ST09H | 116154 | 63122 | 46134 | 25 | 1,87 | 0,2595 | CGCATAGA | CTGAGTCT |
| | ST10H | 77117 | 32037 | 29331 | 24 | 2,429 | 0,4727 | CGCATAGA | GAGGTGAA |
| | ST11H | 115038 | 49838 | 22808 | 21 | 1,996 | 0,3503 | CGCATAGA | GGCATGTA |
| Sed | Sed | 65357 | 35215 | 10585 | 23 | 2,046 | 0,3365 | TCCTGGTA | GTGCAACT |

Table S2. Rank-based Spearman correlation coefficients of the most abundant cyanobacteria ASVs, total cyanobacteria community composition, cyanobacteria 16S rRNA-ITS gene abundance with internal physicochemical parameters in Lake Tiefer See. Values in bold are significant at $p < 0.05$ with Bonferroni *p*-value correction. Spearman correlation coefficients ($R_S$) values highlighted red show a negative correlation, whereas RS values highlighted blue show a positive correlation.

| | Shannon | CCC | 16S rRNA | Turbidity | Temp | pH | Chl-a | DO | $NO_3^-$ | TDP |
|---|---|---|---|---|---|---|---|---|---|---|
| ASV0004_*Aphanizomenon* | 0.24007 | 0.19907 | 0.049033 | **0.39552** | 0.12614 | -0.044474 | 0.065278 | -0.14404 | -0.24663 | 0.17125 |
| ASV0005_*Cyanobium* | 0.1693 | **0.42775** | 0.047298 | -0.10618 | 0.1036 | **-0.35848** | **-0.63782** | **-0.63065** | -0.016639 | 0.31506 |
| ASV0006_*Planktothrix* | 0.022895 | 0.12569 | 0.21751 | 0.029515 | 0.12978 | -0.26648 | -0.23656 | **-0.31275** | 0.10059 | 0.041804 |
| ASV0007_*Synechococcus* | 0.047988 | **0.53885** | 0.17004 | 0.24482 | **0.33239** | 0.25416 | -0.0045136 | 0.065279 | -0.20578 | -0.18739 |
| ASV0008_*Cyanobium* | -0.16536 | -0.049435 | -0.059497 | -0.064425 | -0.11031 | **0.46821** | **0.56957** | **0.68536** | -0.092004 | -0.21856 |
| ASV0009_*Microcystis* | 0.21256 | 0.095783 | -0.064783 | -0.025959 | -0.09784 | -0.017674 | 0.063159 | -0.010528 | 0.078191 | -0.031918 |
| ASV0014_*Cyanobium* | **0.37755** | 0.20849 | **-0.29517** | **0.30681** | -0.10762 | **-0.51783** | **-0.55132** | **-0.64223** | 0.30912 | **0.49326** |
| ASV0016_*Snowella* | **0.37502** | **0.3778** | -0.010639 | 0.28227 | 0.21315 | -0.11515 | -0.23475 | **-0.38262** | -0.0049261 | 0.10764 |
| Shannon diversity | | **-0.3262** | -0.2148 | -0.20678 | -0.14929 | -0.088712 | -0.19854 | -0.072275 | 0.17765 | 0.2941 |
| CCC | -0.3262 | | 0.24856 | **0.33644** | **0.49991** | 0.09461 | -0.07326 | -0.24741 | **-0.36219** | -0.15182 |
| 16S rRNA-ITS abundance | -0.2148 | 0.24856 | | 0.085416 | **0.74694** | **0.32438** | **0.32424** | 0.031744 | **-0.36927** | **-0.46901** |

Table S3 Similarity percentage (SIMPER) test on the cyanobacteria ASVs among the different sample types. Only ASVs with > 1% relative abundance in the sample types were used in the for the test.

| Taxon | ASV_ID | Av. sim | Contrib. % | Cumulative % | Mean Water | Mean TrapM | Mean TrapH | Mean Sed |
|---|---|---|---|---|---|---|---|---|
| Aphanizomenon sp. | ASV_0004 | 4.427 | 9,34 | 9.34 | 1.01 | 3.64 | 4 | 5.04 |
| Cyanobium sp. | ASV_0008 | 3.203 | 6.758 | 16.1 | 2.72 | 0.852 | 0.483 | 0.558 |
| Planktothrix sp. | ASV_0006 | 2.526 | 5.328 | 21.43 | 2.51 | 0.586 | 0.752 | 0.541 |
| Synechococcus sp. | ASV_0007 | 2.374 | 5.008 | 26.43 | 3.11 | 1.98 | 1.51 | 1.01 |
| Cyanobium sp. | ASV_0005 | 2.258 | 4.765 | 31.2 | 2.37 | 2.02 | 1.89 | 1.61 |
| Cyanobium sp. | ASV_0013 | 2.222 | 4.689 | 35.89 | 2.4 | 0.865 | 0.747 | 0.74 |
| Snowella sp. | ASV_0016 | 2.219 | 4.681 | 40.57 | 0.906 | 1.88 | 2.5 | 1.02 |
| Cyanobium sp. | ASV_0010 | 2.151 | 4.538 | 45.11 | 1.77 | 2.42 | 2.22 | 1.9 |
| Cyanobium sp. | ASV_0012 | 1.969 | 4.154 | 49.26 | 1.42 | 2.27 | 1.92 | 1.65 |
| Cyanobium sp. | ASV_0015 | 1.948 | 4.109 | 53.37 | 2.12 | 1.1 | 0.896 | 0.68 |
| Cyanobium sp. | ASV_0014 | 1.898 | 4.004 | 57.37 | 1.4 | 1.3 | 2.41 | 1.12 |
| Cyanobium sp. | ASV_0019 | 1.83 | 3.861 | 61.24 | 1.77 | 0.426 | 0.445 | 0 |
| Synechococcus sp. | ASV_0021 | 1.662 | 3.507 | 64.74 | 1.66 | 0.323 | 0.456 | 0.599 |
| Cyanobium sp. | ASV_0031 | 1.553 | 3.277 | 68.02 | 1.6 | 0.523 | 0.499 | 0 |
| Snowella sp. | ASV_0022 | 1.553 | 3.276 | 71.3 | 0.786 | 1.35 | 1.64 | 0.476 |
| Cyanobium sp. | ASV_0020 | 1.446 | 3.051 | 74.35 | 1.09 | 1.79 | 1.67 | 3.99 |
| Aphanizomenon sp. | ASV_0035 | 1.433 | 3.024 | 77.37 | 0.478 | 1.29 | 1.39 | 2 |
| Cyanobium sp. | ASV_0053 | 1.314 | 2.772 | 80.14 | 1.07 | 0.0541 | 0.0572 | 0.292 |
| Cyanobium sp. | ASV_0049 | 1.258 | 2.653 | 82.8 | 0.434 | 1.12 | 1.23 | 0.987 |
| Planktothrix sp. | ASV_0050 | 1.158 | 2.443 | 85.24 | 0.903 | 0 | 0.0651 | 0 |
| Microcystis sp. | ASV_0009 | 1.077 | 2.272 | 87.51 | 0.724 | 0.644 | 1.02 | 1.59 |
| Cyanobium sp. | ASV_0030 | 1.06 | 2.236 | 89.75 | 1.47 | 0.909 | 0.78 | 0.364 |
| Cyanobium sp. | ASV_0028 | 1.049 | 2.213 | 91.96 | 1.04 | 0.863 | 1.26 | 1.15 |
| Cyanobium sp. | ASV_0056 | 0.9107 | 1.921 | 93.88 | 0.941 | 0.236 | 0.379 | 0 |
| Cyanobium sp. | ASV_0054 | 0.8186 | 1.727 | 95.61 | 0.699 | 0.64 | 0.811 | 0.466 |
| Microcystis sp. | ASV_0041 | 0.7399 | 1.561 | 97.17 | 0.647 | 0.961 | 1.03 | 0.659 |

Table S4 Total sulphide measurements from water column samples.

| Date | Water Depth [m] | Sample ID | Observation | H2S_µmolL-1 |
|---|---|---|---|---|
| 30.10.2019 | 54 | 54 | filtered | 2 |
| 30.10.2019 | 55 | 55a | filtered | 7,9 |
| 30.10.2019 | 55 | 55b | filtered | 5,2 |
| 30.10.2019 | 57 | 57 | filtered | 33,3 |
| 30.10.2019 | 59,5 | 59,5 | filtered | 44,8 |
| 30.10.2019 | 60,8 | 60,8 | filtered | 42,5 |
| 28.11.2019 | 53 | 53 | filtered | 11 |
| 28.11.2019 | 55 | 55 | filtered | 13,7 |
| 28.11.2019 | 57 | 57 | filtered | 9,7 |
| 28.11.2019 | 59 | 59 | filtered | 34 |

Table S5 Nitrate measurements from water column samples. NA = not ascertained.

| SampleID | $NO_3^-$ |
|---|---|
| WMay2_3 | 0,57 |
| WMay29_3 | 0,14 |
| WJu27_3 | 0,19 |
| WAug6_3 | *NA* |
| WAug28_3 | *NA* |
| WOc2_3 | *NA* |
| WOc30_3 | *NA* |
| WNo28_3 | 0,30 |
| WJa30_5 | 2,02 |
| WFe24_5 | 1,81 |
| WMar28_5 | 1,40 |
| WMay2_7 | 1,01 |
| WMay29_7 | 1,04 |
| WJu27_7 | 1,52 |
| WAug6_7 | *NA* |
| WAug28_7 | *NA* |
| WOc2_7 | *NA* |
| WOc30_7 | *NA* |
| WNo28_7 | 0,19 |
| WMar28_10 | 1,38 |
| WMay2_10 | 1,26 |
| WMay29_10 | 2,51 |
| WJu27_10 | 1,94 |
| WAug6_10 | 1,71 |
| WAug28_10 | 1,08 |
| WOc2_10 | 0,47 |

| | |
|---|---|
| WOc30_10 | *NA* |
| WNo28_10 | 0,20 |
| WMay2_15 | 1,64 |
| WMay29_15 | 1,85 |
| WJu27_15 | 2,07 |
| WAug28_15 | 2,04 |
| WOc2_15 | 2,31 |
| WOc30_15 | 2,24 |
| WNov28_15 | 2,00 |
| WMar28_20 | 2,16 |
| WAug6_20 | 2,14 |
| WJa30_45 | 1,96 |
| WFe24_45 | 1,74 |
| WMar28_45 | 1,71 |
| WMay2_45 | 1,85 |
| WMay29_45 | 2,02 |
| WJu27_45 | 1,96 |
| WAug6_45 | 2,11 |
| WAug28_45 | 1,73 |
| WOc2_45 | 2,04 |
| WOc30_45 | 0,90 |
| WNo28_45 | 0,75 |

Table S6

ANOVA test for equal means on species richness from sediments and water column ($P < 0.05$; Figure 4). A subsequent Tukey's pairwise test showed hypolimnion sediment trap cyanobacteria communities to significantly differ to the communities from the epi-, meta and hypolimnion communities of the water column. Tukey's Q below the diagonal and P-values above the diagonal. Significant P-values are in bold. Shapiro Wilk: 0.9915, p_normal:0.9447. Where 'W' indicates water column samples from the from the epi-, meta- and hypolimnion, respectively. The 'ST' indicates sediment trap material from the meta- and hypolimnion, respectively. 'Sed' represents the surface sediment samples.

| **ANOVA Test for equal means across sample types** | | | | | |
|---|---|---|---|---|---|
| | Sum of sqrs | df | Mean square | F | p (same) |
| Between groups: | 1007.74 | 4 | 251.935 | 6,133 | 0.006964 |
| Within groups: | 3279.15 | 58 | 64.2957 | Permutation p (n=99999) | |
| Total: | 4736.69 | 62 | 0.00627 | | |

| **Tukey's pairwise** | W_Epi | W_Meta | W_Hypo | ST_Meta | ST_Hypo |
|---|---|---|---|---|---|
| W_Epi | | 0.9989 | 0.9982 | 0.2364 | **0.03609** |
| W_Meta | 0.3772 | | 1 | 0.2609 | **0.04405** |
| W_Hypo | 0.4292 | 0.03314 | | 0.1508 | **0.01942** |
| ST_Meta | 2.962 | 2.884 | 3.293 | | 0.9518 |
| ST_Hypo | 4.165 | 3.927 | 4.496 | 1.014 | |

Table S7 The significant explanatory parameters used in the Spearman correlation analysis.

| **Explanatory variable** | **AIC** | **Pseudo-*F*** | ***P*** |
|---|---|---|---|
| DO | 159.73 | 8.77 | 0.001 |
| Turbidity | 157.26 | 4.92 | 0.002 |
| TDP | 154.76 | 4.56 | 0.002 |
| pH | 153.98 | 4.42 | 0.005 |
| $NO_3$- | 152.98 | 3.56 | 0.004 |
| Temperature | 150.95 | 3.07 | 0.008 |

The significance of the environmental variables was tested by 999 Monte Carlo permutations and were selected by forward selection (Adjusted $R^2$ = 0.34). AIC = Akaike information criterion.
